# Housing quality and school outcomes in England: a nationally representative linked cohort study

**DOI:** 10.1136/jech-2025-224495

**Published:** 2025-12-16

**Authors:** Gergő Baranyi, Katie Harron, Sierra N Clark, Emla Fitzsimons

**Affiliations:** 1 Centre for Longitudinal Studies, UCL Institute of Education, University College London, London, UK; 2 Population, Policy & Practice Department, UCL GOS Institute of Child Health, University College London, London, England, UK; 3 Department of Population Health & Policy, School of Health & Medical Sciences, City St George’s University of London, London, England, UK; 4 Institute for Fiscal Studies, London, UK

**Keywords:** HOUSING, EDUCATION, COHORT STUDIES, LONGITUDINAL STUDIES, CHILD HEALTH

## Abstract

**Background:**

One in seven households in England live in accommodation not meeting housing quality standards. Low-quality housing is linked to adverse child health, but less is known about the relationship with educational outcomes. This study evaluated the relationship between housing quality, school absences and educational attainment.

**Methods:**

Data were drawn from the Millennium Cohort Study, a nationally representative cohort of children born in 2000/2002. Housing quality at age 7 years was computed from six indicators: accommodation type, floor level, access to a garden, damp, heating and overcrowding. Percentage of missed school sessions and standardised test scores in Maths and English at age 7, 11 and 16 were linked from the National Pupil Database. Confounder-adjusted linear regressions with survey weights were fitted.

**Results:**

Approximately 16% of children lived in lower quality housing (ie, disadvantage in ≥2 conditions); after confounder adjustment, these children had 0.74% (or 1.4 days) more absences per year than those living in higher quality housing (n=7272, 95% CI 0.34% to 1.13%). Damp, overcrowding and accommodation type were the strongest predictors of absence. Test scores in Maths and English across compulsory schooling were between 0.07 and 0.13 SD lower for children living in lower versus higher quality housing (n=6741), mainly driven by overcrowding and lack of central heating.

**Conclusion:**

Children living in homes with lower quality housing conditions missed 15.5 days more of school throughout compulsory schooling and performed worse on national tests than those in higher quality housing. Targeting specific housing conditions, such as damp and overcrowding, could be beneficial for children’s school outcomes.

**WHAT IS ALREADY KNOWN ON THIS TOPIC**

- Housing is a key determinant of child health. However, relatively little is known about how housing quality relates to educational outcomes, particularly school absences and educational attainment, and existing research often focuses on single housing conditions only.

**WHAT THIS STUDY ADDS**

- Using a nationally representative sample of English pupils, this comprehensive study found that lower housing quality was associated with increased school absences over the course of compulsory schooling (15.5 additional days over 11 years) and lower performance in national exams during both primary and secondary school. Damp, overcrowding and living in flats were linked to greater school absence. Overcrowding and lack of central heating were associated with lower academic attainment.

**HOW THIS STUDY MIGHT AFFECT RESEARCH, PRACTICE OR POLICY**

- Improving housing conditions, specifically by reducing overcrowding and damp, and upgrading heating systems, may benefit both children’s health and their educational outcomes. Future research should prioritise objective, systematic measurement of the indoor home environment and explore the long-term consequences of poor housing quality.

## Introduction

 People spend the majority of their time at home^1^; however, in England, one in seven homes fail to meet the Decent Homes Standard (ie, safety, repair, modern facilities and services, and adequate heating).^2^ Furthermore, 3% of homes are overcrowded and 4% have severe cases of any type of damp.^2^ Housing is a well-established social determinant of health^3^,^6^; beyond financial aspects (eg, affordability, residential stability), indoor environmental quality and characteristics, such as overcrowding, damp and mould, air pollution and chemical exposures, noise and thermal comfort, all play a significant role in influencing health and exacerbating health inequalities. Socioeconomic and environmental conditions are also intertwined. In England, poor housing conditions are more common among the most disadvantaged,^7^ including ethnic minorities.^2^ For example, Black households are three times more likely (12%) to live in homes with damp problems compared with White households (4%).^2^

Children may be more exposed to,^2^ and particularly vulnerable to, risks associated with substandard housing.^4 5^ For example, in England, ~7300 annual new cases of asthma and lower respiratory infections among children were attributable to damp and/or mould in 2019.^8^ Housing might play a role in educational outcomes too. In addition to having less suitable places for study, children affected by asthma or other health conditions miss school more often^9 10^ and are less attentive, which can lead to lower grades.^11^ However, research on housing and educational outcomes remains limited, especially for school absences.^12^ Moreover, there is a lack of evidence on the specific housing conditions that are most strongly associated with child outcomes.^11^

This study examined the relationship between housing quality, school absences and educational attainment by linking English longitudinal cohort data with educational administrative records throughout compulsory schooling. In addition to assessing overall housing quality, specific conditions were also analysed and effect modification by sex and household income explored.

## Methods

Data were drawn from the Millennium Cohort Study (MCS), a large and nationally representative cohort of approximately 19 000 children born in 2000/2002 in the UK.^13 14^ The sample was recruited at the age of 9 months using government child benefit records, with disproportionate representation for ethnic minority and disadvantaged populations.^13^ Following the recruitment, follow-up waves (ie, sweeps) were conducted at age 3, 5, 7, 11, 14 and 17 years. At age 7, caregivers were asked to provide written consent to link cohort members’ educational data up to age 16.^15^ For consented pupils residing in England (93.8% consented), administrative records from the National Pupil Database, curated by the Department for Education, were linked with a success rate of 99.4%.^15 16^ This study included MCS participants residing in England with linked educational data.

### Housing quality

Housing quality was assessed at age 7 using a modified scale from Pearce *et al*.^17^ Six conditions were derived from information provided by the main caregiver and classified into positive (0), intermediate (1) and negative (2) features: accommodation type (0=house; 1=flat; 2=room, or other), floor level (0=ground floor; 1=1st floor; 2=basement/2nd floor or above), having access to a garden (0=sole access; 1=shared access; 2=no access) (ie, also referred to as a ‘back-yard’ in some places), damp (0=no damp; 1=not much of a problem/some problem; 2=great problem), heating (0=central heating; 2=other types of heating) and overcrowding (0=2+ rooms per person; 1=1 to <2 rooms per person; 2=<1 room per person). As the first three items were only collected when the family moved, missing information was imputed from previous waves. Item responses were summed up, creating a housing quality score (0–12) with higher values indicating lower quality. We also computed a binary indicator (*higher* vs *lower*) using the cut-off value of ≥3, suggesting disadvantage in at least two conditions.

### Educational records

Two sets of information^18^ were derived from the linked National Pupil Database comprising data from all state-funded schools in England.^19^

1. *School absence*: For each academic year (2006/2007 (Year 1)–2016/2017 (Year 11)), the percentage of sessions missed due to authorised (ie, permitted by school representative), unauthorised (ie, not permitted, late arrivals) and total (ie, aggregate of authorised and unauthorised) absence were computed based on the number of possible and missed sessions.^20^ There is a morning and afternoon session each day, a minimum of 380 per year. The academic year is made up from autumn, spring and summer terms, each separated into two half-terms.^20^ Until 2012/2013, the Department for Education did not collect absence data for the second summer (or sixth) half term^20^; to maintain comparability across the study, attendance during the first five half terms was used.^18^
2. *Attainment*: State-funded schools follow the national curriculum across four key stages (KS): KS1 (Year 1–2/age 5–7), KS2 (Year 3–6/age 7–11), KS3 (Year 7–9/age 11–14) and KS4 (Year 10–11/age 14–16).^21^ Test scores for English and Maths at the end of KS1, KS2 and KS4, or at ages 7, 11 and 16 years, respectively, were used to assess performance. Teacher-administered English Reading and Maths test scores from KS1, national test marks for English and Maths from KS2, and English and Maths GCSE (General Certificate of Secondary Education) scores from KS4 were used. Attainment 8 (A8), a composite score across eight GCSE-level qualifications with double-weighted English and Maths, was also included. Test scores were standardised to support effect size comparison, and we dropped participants (<1%) if their exams did not take place in the same academic year as for the rest of the sample.^18^

### Covariates

Confounders were identified using a directed acyclic graph (online supplemental figure S1), derived from the earliest available sweep, and included sex (female; male), month of birth, ethnic groups (Black or Black British; Indian; Pakistani and Bangladeshi; White; mixed; other ethnic groups), main caregiver’s partnership status (single parent; living with partner), highest household educational attainment and household income quintiles. Education was assessed using the National Vocational Qualification (NVQ) scale (none/unknown; overseas only; NVQ1–NVQ5), and the highest value in the household was taken. Household income was categorised into five groups using the Organisation for Economic Co-operation and Development income-weighted quintiles.

### Statistical analysis

Linear regression models were fitted to estimate the associations between housing quality, school absence and educational attainment. To compensate for design-related oversampling and attrition—thus, to ensure that findings were representative—survey weights were applied in all analyses. Analyses were conducted using the *survey*^22^ package in R 4.3.0.^23^

Primary analyses explored associations with overall housing quality. Unadjusted and adjusted associations were presented. Coefficients (*b*) were expressed in standard deviation (SD) increase in the housing quality score and presented with their 95% confidence intervals (95% CI). In the secondary analyses, we showed adjusted associations for each housing condition separately; coefficients were expressed as value increase (ie, from positive to intermediate, from intermediate to negative feature). For absence, in addition to year-specific associations, pooled analyses with robust standard errors (ie, annual data stacked into one long-formatted database with year added as a further covariate) were fitted to estimate the average association with overall absence across compulsory schooling.

We explored effect modification by sex and household income when entering the study, by adding an interaction term to the adjusted models; overall p values were determined using the Wald test. To reduce type I error due to the large number of hypotheses testing, we presented false discovery rate adjusted p values (Benjamini and Hochberg procedure) for school absence.^24^

Six sensitivity analyses were carried out. First, as a significant proportion of participants moved house between sweeps (eg, 10% between age 5 and 7), we restricted the sample to those who stayed at the same address as reported at the time of exposure assessment. Second, missing data were imputed using multiple imputation by chained equations; 10 datasets were pooled using Rubin’s rules. Third, to provide comparable estimates across specific housing conditions, we re-ran analyses with standardised items. Fourth, because the percentage of missed sessions was left-skewed (see online supplemental figure S2), we also fitted models with log-transformed absence data. As the results were similar, we reported models based on untransformed values as the main analysis to aid interpretation and included the transformed ones as sensitivity. Fifth, we presented housing condition-specific analyses by treating variables as factors (positive value as reference). Last, instead of pooling 11 years of absence data, we analysed them longitudinally using mixed-effects linear regression with random intercept (ie, observations nested within individuals).

## Results

**Figure 1:**
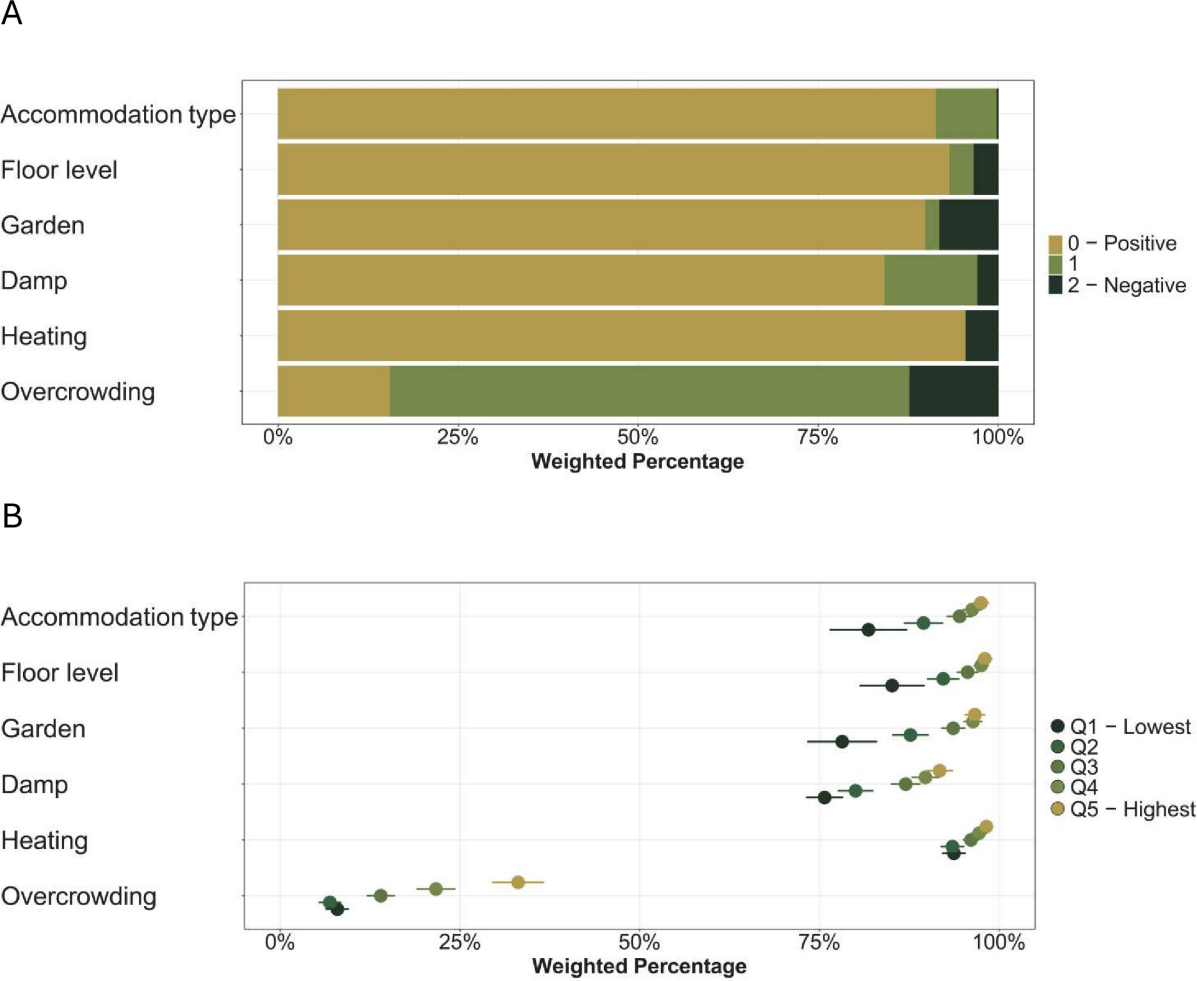
Weighted percentage of participants (A) with different features for each housing condition and (B) the weighted percentage of participants with positive features only across income quintile groups. Figures are based on the *Absence sample* with a sample size of n=7272.

Out of 8992 participants living in England at age 7, caregivers’ consent for educational data linkage was available for 8437. To minimise loss of data due to missing records, we created separate samples for absence (n=7272) and attainment (n=6741) (online supplemental figure S3). Participants in the analytical samples were similar to the English sample representing the population, being somewhat more likely White and belonging to middle household income/education groups (table 1). Approximately 84% of the sample lived in accommodation in higher housing quality (0–2). The percentage of children exposed to different levels of housing conditions is shown in figure 1A (frequencies in online supplemental table S1); pupils from households with lower income were more likely to live in lower-quality housing (figure 1B). Correlation between type of accommodation, floor level and access to garden was high (Kendall’s *τ*>0.68), while damp, heating and overcrowding had low correlation with all other conditions (Kendall’s *τ*<0.16) (online supplemental figure S4).

**Table 1:** Weighted sample characteristics

	English sample* (n=8992)	Absence sample (n=7272)	Attainment sample (n=6741)
Sex, % (n)			
Female	48.6 (4441)	48.8 (3594)	50.0 (3408)
Male	51.4 (4545)	51.2 (3678)	50.0 (3333)
Ethnic group, % (n)			
Black or Black British	3.9 (444)	3.4 (313)	3.5 (301)
Indian	2.3 (335)	2.1 (255)	2.1 (245)
Mixed	3.9 (346)	3.6 (260)	3.4 (228)
Pakistani and Bangladeshi	5.5 (848)	5.0 (651)	5.2 (609)
White	82.9 (6764)	84.4 (5659)	84.4 (5230)
Other ethnic groups	1.5 (170)	1.5 (134)	1.5 (128)
Partnership status, % (n)			
Single parent	15.6 (1283)	15.8 (1052)	14.2 (882)
Living with partner	84.4 (7692)	84.2 (6220)	85.8 (5859)
Highest household education, % (n)			
No education	9.9 (905)	9.9 (725)	8.5 (597)
Overseas only	2.1 (236)	2.1 (195)	2.0 (171)
NVQ level 1	6.3 (537)	6.7 (464)	6.2 (397)
NVQ level 2	25.8 (2229)	27.9 (1960)	27.8 (1804)
NVQ level 3	15.4 (1350)	16.1 (1140)	16.4 (1068)
NVQ level 4	34.3 (3111)	32.5 (2411)	34.1 (2333)
NVQ level 5	6.3 (612)	4.7 (377)	5.0 (371)
Income quintiles, % (n)			
Q1 – lowest income quintile	21.2 (1952)	21.8 (1619)	19.6 (1370)
Q2	20.9 (1966)	21.9 (1659)	21.4 (1511)
Q3	20.0 (1716)	21.2 (1485)	21.8 (1415)
Q4	18.9 (1661)	19.4 (1394)	20.5 (1354)
Q5 – highest income quintile	19.0 (1658)	15.7 (1115)	16.7 (1091)
Housing quality score (0–12)			
Mean±SD	1.6±1.6	1.6±1.6	1.6±1.5
Median (IQR)	1 (1–2)	1 (1–2)	1 (1–2)
Housing quality, % (n)			
Higher (0–2)	83.5 (7359)	83.3 (6055)	84.2 (5655)
Lower (3–12)	16.5 (1493)	16.7 (1217)	15.8 (1086)

Month of birth is not included in the table but was included in models. Means and percentages are weighted using complex sample weights; frequencies are unweighted. Percentages might not add up to 100 due to rounding error.

* Frequencies might not add up to total due to missing values.

NVQ, National Vocational Qualification.

### School absence

**Figure 2:**
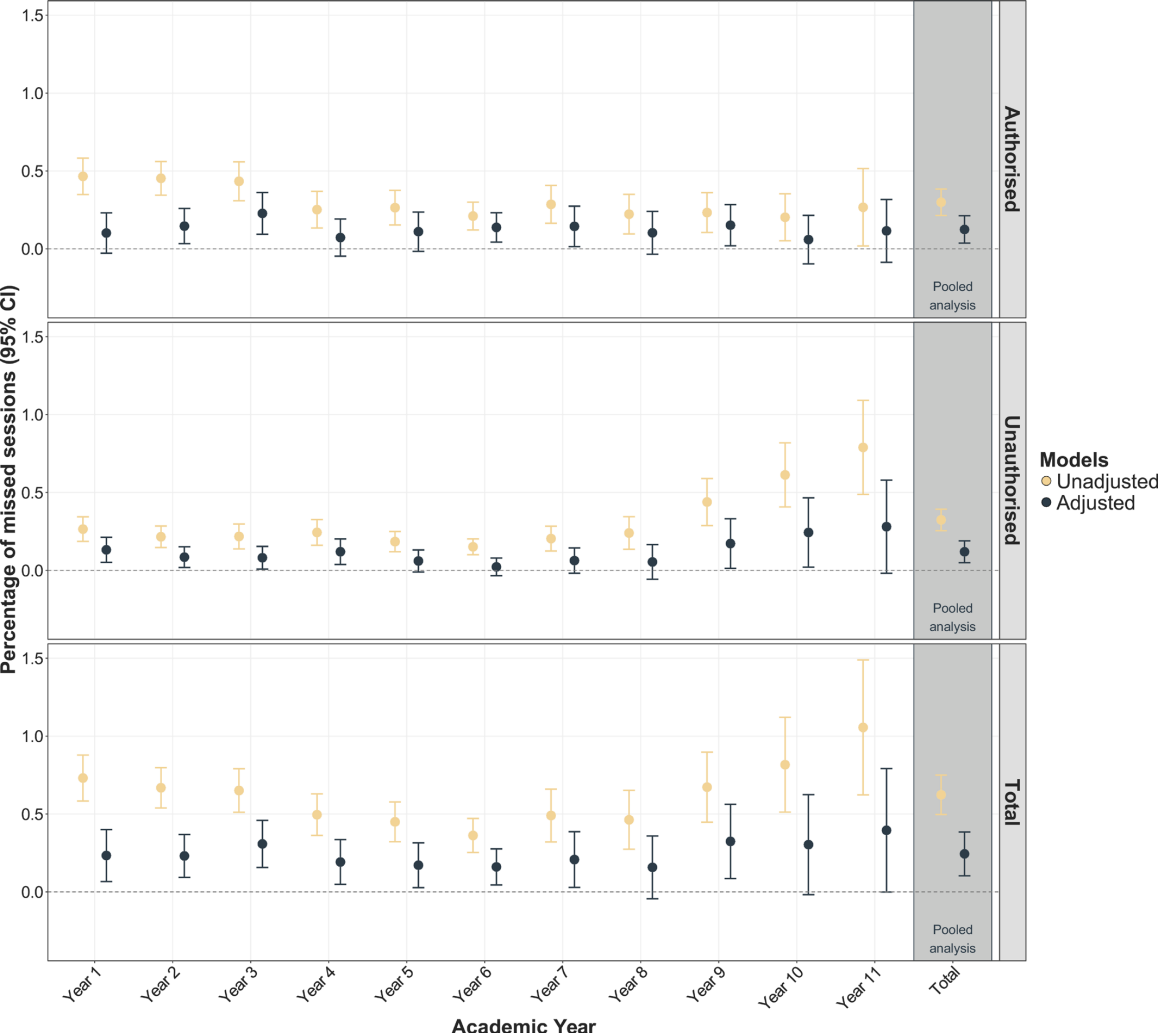
Difference in % of missed sessions for each 1SD decrease in the housing quality score. Linear regressions with complex survey weights were fitted; adjusted models controlled for sex, month of birth, ethnic groups, maternal partnership status, highest household education and household income. Housing quality score was standardised; the sample size was n=7272.

On average, children missed 5.1% of the total sessions (86 days) during compulsory schooling (Years 1–11); 4.9% within higher and 6.5% within lower quality housing, respectively (online supplemental table S2). In the unadjusted models, lower quality housing was associated with higher percentage absence in every academic year (figure 2). After adjusting for confounders, effect estimates attenuated but remained statistically significant for total absences and mainly during primary school (ie, Years 1–6). Each 1SD increase in the housing quality score was associated with 0.24% (95% CI 0.10% to 0.38%) more missed sessions during Years 1–11; 0.12% (95% CI 0.04% to 0.21%) due to authorised and 0.12% (95% CI 0.05% to 0.19%) due to unauthorised absence (figure 2, online supplemental table S3).

Based on the dichotomised housing quality indicator, in comparison to higher-quality housing, lower-quality housing was linked to a 0.74% (95% CI 0.34% to 1.13%) increase in total absences during Years 1–11 (online supplemental table S4), which corresponds to a difference of 31 more sessions (or 15.5 days; extrapolated based on six half terms) missed during compulsory schooling.

**Figure 3:**
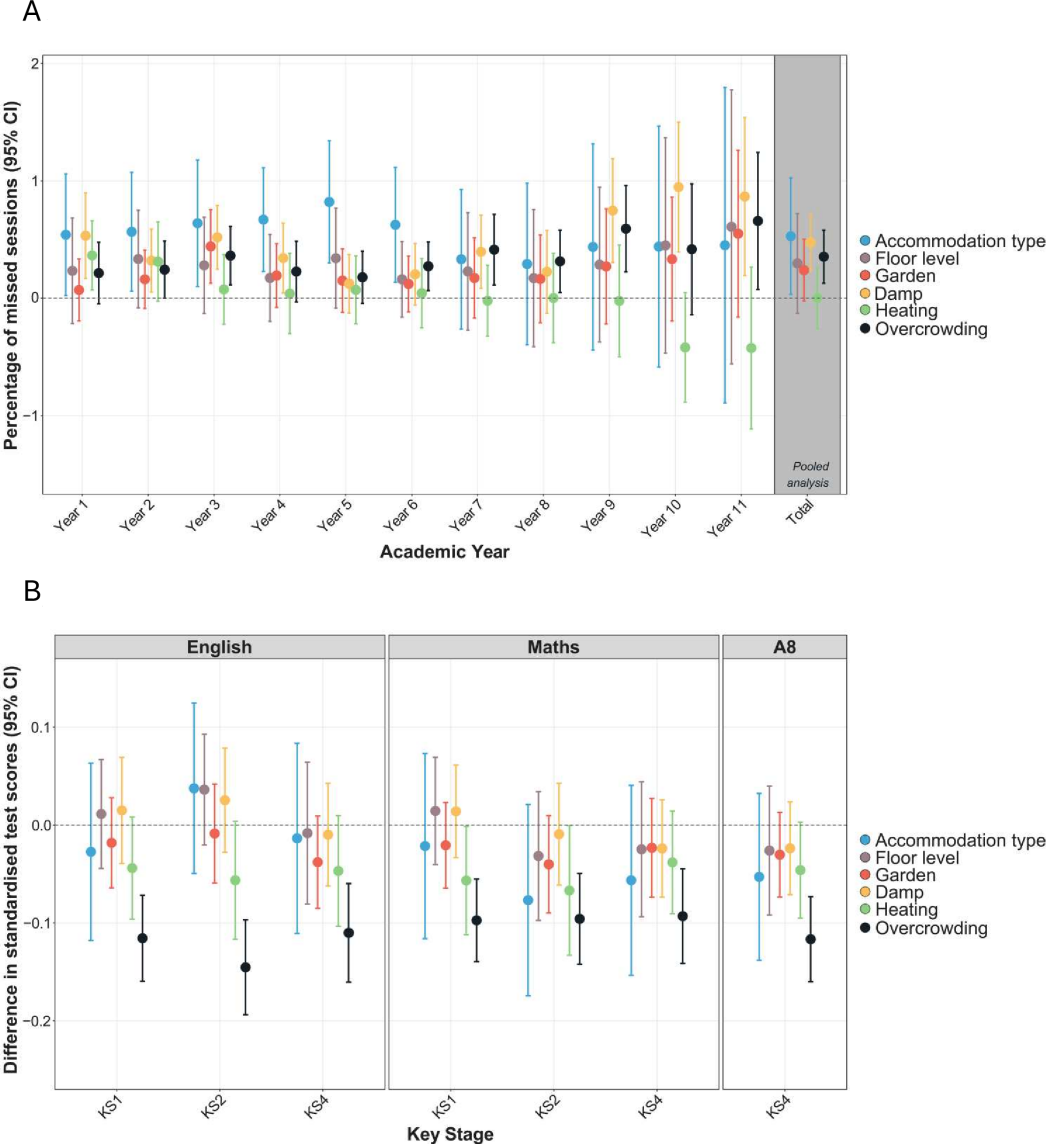
Negative housing conditions and (A) difference in % of missed sessions due to total school absence and (B) difference in educational attainment. Linear regressions with complex survey weights were fitted; adjusted models controlled for sex, month of birth, ethnic groups, maternal partnership status, highest household education and household income. The sample size was n=7272 for A, and n=6741 for B.

Housing condition-specific analyses showed that accommodation type (b=0.53), damp (b=0.47) and overcrowding (b=0.35) were associated with total school absence (figure 3A; online supplemental table S5). Authorised absence was associated with damp (b=0.33) and accommodation type (b=0.32), unauthorised with restricted access to garden (b=0.20), overcrowding (b=0.20) and damp (b=0.14) (online supplemental tables S6 and S7).

### Educational attainment

Lower quality housing was associated with lower exam performance (table 2, online supplemental table S8). After adjusting for confounders, effect estimates uniformly attenuated by 70–80% but remained significant (apart from KS2 English) (table 2). Each 1SD increase in the housing quality score was associated with a 0.03 SD decrease in KS1 English and Maths, and a 0.04 SD decrease in KS2 Maths, and KS4 English, Maths and A8 scores (table 2).

**Table 2:** Difference in educational attainment (ie, standardised test scores) for each 1SD increase in the housing quality score

	Unadjusted			Adjusted		
	β	95% CI	P value	β	95% CI	P value
Key stage 1						
English (Reading)	−0.14	−0.16 to 0.11	<0.001	−0.03	−0.05 to 0.00	0.022
Maths	−0.12	−0.15 to 0.10	<0.001	−0.03	−0.05 to 0.00	0.032
Key stage 2						
English	−0.12	−0.15 to 0.09	<0.001	−0.02	−0.05 to 0.00	0.060
Maths	−0.12	−0.15 to 0.09	<0.001	−0.04	−0.07 to 0.01	0.004
Key stage 4						
English	−0.13	−0.16 to 0.10	<0.001	−0.04	−0.06 to 0.01	0.010
Maths	−0.14	−0.17 to 0.11	<0.001	−0.04	−0.06 to 0.01	0.012
Attainment 8	−0.14	−0.17 to 0.11	<0.001	−0.04	−0.07 to 0.02	0.001

Linear regressions with complex survey weights were fitted. Adjusted models controlled for sex, month of birth, ethnic groups, maternal partnership status, highest household education and household income. The sample size was n=6741.

Using the dichotomised housing quality measure, children living in lower-quality housing had test scores that were between 0.07 and 0.13 SD lower than those living in higher-quality housing (online supplemental table S9).

Housing condition-specific analyses showed that overcrowding was significantly associated with all test scores, with estimates ranging between −0.09 (KS4 Maths) and −0.15 (KS2 English). We also found some associations between not having central heating and lower KS1 (b=−0.06) and KS2 (b=−0.07) Maths test scores (figure 3B; online supplemental table S10).

### Explorative and sensitivity analyses

We did not find effect modification by income or sex: the association between housing quality and absence and attainment did not differ across these groups (online supplemental tables S11 and S12).

Sensitivity analyses reinforced the main findings. First, after restricting the sample to non-movers, housing quality was still associated with school absence during primary school (online supplemental table S13). Findings for educational attainment remained comparable, with KS2 and KS4 English not associated with housing quality (online supplemental table S14). Second, multiple imputation did not change the results (online supplemental tables S15 and S16). Third, standardising the housing condition variables suggested that damp had the strongest link to school absence (online supplemental tables S17 and S18). Fourth, analysing log-transformed absence variables showed similar associations as presented in the main analyses (online supplemental tables S19 and S20). Fifth, analysing housing conditions as factors (ie, values 0 (reference), 1, 2) demonstrated that living in a flat (vs a house), having some and great problems with damp (vs none), having access to a shared garden (vs sole use), and residing in overcrowded homes (<1 room per person vs 2+ rooms per person) increased school absence (online supplemental table S21). In comparison to no overcrowding, living in homes with 1 to <2 rooms per person, and especially residing in 2=<1 room per person homes, was associated with lower test scores in all national exams (online supplemental table S22). Lastly, analysing absence data longitudinally (ie, mixed effects regression) showed comparable findings for the housing score, and for damp and overcrowding (online supplemental table S23).

## Discussion

This study found that children in lower quality housing had higher school absences, especially during primary school, and lower educational attainment in national exams, compared with children living in higher quality housing. The presence of damp, overcrowding and living in flats contributed to higher school absences. School performance was almost exclusively associated with overcrowding, with some findings showing lower primary school Maths scores when living in accommodations without central heating.

At age 7, 16–17% of our cohort lived in lower quality housing (as defined by having a combined score of ≥3) which was linked to 0.74% more sessions missed during compulsory schooling (or 1.4 additional days annually); associations with absence were more robust during primary school. Given that in 2016/2017 (ie, the year when MCS participants finished compulsory schooling) English pupils missed 8 school days on average,^25^ this is a notable difference. In England, 84% of authorised absences are health-related (medical appointments or sickness).^25^ Damp homes have been associated with respiratory conditions and exacerbation of conditions among children,^26 27^ which can be a factor contributing to higher school absences.^10^ Indoor dampness is a determinant of allergens, respiratory irritants and infectious agents, such as dust mites and microbes, and can enhance emissions of volatile organic compounds. While study participants were not asked directly about the presence of mould, damp conditions are a major determinant of fungal growth which can in turn release hazardous spores, fragments and microbial volatile organic compounds into indoor air.^28^ Housing conditions could also have an impact on health via a stress-response pathway: children in substandard housing (eg, low cleanness, overcrowding) have higher cortisol levels,^29^ and there is correlational evidence between damp and mould and psychological symptoms.^5^

Overcrowding in early life can have life-long implications.^30^ Crowded housing is linked to behavioural problems and to worse health in children.^31^ In comparison to houses, flats are more often overcrowded in England,^32^ and they more often have damp^2^ and other hazardous exposures^7^ leading to a higher risk of microbial and chemical exposures, and easier contagion of infectious diseases, which can increase school absence due to sickness. Overcrowding can also lead to behavioural problems through maternal stress, strained parental–child interactions and less sleep,^33^ which might explain its association with unauthorised absence.

Living in crowded homes is linked to lower academic achievement due to noise, lack of study space, insufficient sleep, reduced concentration and added responsibilities (eg, childcare).^30 31 34^ Our findings showed that home overcrowding was linked to lower test scores across almost all exams, aligning with prior studies from the USA^31^ and Latin America.^34^ Overcrowding during high school has also been linked to lower graduation rates and educational attainment by the age of 25.^30^ We found associations between the absence of central heating (eg, no heating, coal, gas or electric fires) and lower Maths scores in primary school, likely due to colder indoor temperatures, which is a risk factor of damp but can also directly reduce cognitive performance.^35^

Improving housing conditions, especially reducing damp and overcrowding, and updating heating systems and energy efficiency can have significant benefits.^36^ Given the magnitude of the problem in England,^2^ national and local public health and housing policies targeting these features of housing quality could improve children’s health and school outcomes across the country and narrow the health inequality gaps. There are likely cost savings; it costs £1.4 (€1.6, US$1.8) billion each year for the NHS to treat people affected by poor housing.^37^ Nationally, the government is taking steps to improve awareness and knowledge of the health risks of damp and mould among landlords and housing providers with the publication of new national guidance.^38^ Additionally, Awaab’s law came into effect in October 2025, forcing social landlords to investigate and fix dangerous damp and mould, and repair all emergency hazards within set time periods.^39^ As poorer housing conditions are more common among lower-income households, where children more often miss school and have lower educational attainment,^18^ interventions would particularly benefit disadvantaged pupils and likely contribute to narrowing health and educational inequalities, and better future labour market outcomes.

### Strengths and limitations

This longitudinal study following children across compulsory schooling linked high-quality administrative data^19^ to a rich and multidisciplinary birth cohort,^13^ thus taking advantage of both types of data and reducing common method bias. Findings are representative and generalisable in the age-specific English population educated in state-funded schools. There are limitations to consider. First, despite the high consent and overall linkage success rate, 10% of participants had missing absence or attainment data for at least one data point. An additional 7% of participants did not sit exams and they had lower-quality housing (mean score of 1.8 vs 1.6) suggesting that the negative effects on attainment are likely underestimated. Children exclusively in home schooling and private schools (7%) are not captured in the National Pupils Database.^19^ Second, housing conditions were measured as parental report and not investigated using objective measures. We applied a threshold of ≥3 to identify lower housing quality; other cut-offs might lead to different findings. Third, several key aspects of housing (eg, indoor air pollution, noise, thermal comfort, lighting and pests) were not captured. Fourth, we assessed the home environment at age 7, as some housing conditions were not measured consistently across the follow-up (eg, damp), and age 7 data provided the largest sample size and best proximity to outcomes. Excluding participants who moved during the study confirmed the robustness of our findings. Fifth, absence data are based on the first five half terms, and children miss slightly more school during the sixth half term.^20^ Sixth, although we conducted a sensitivity analysis using log-transformed absence data that confirmed the main findings, we cannot exclude the possibility that other assumptions of linear regression were violated. Last, although models were adjusted for key confounders, we cannot rule out residual confounding. 

## Conclusions

In a nationally representative and longitudinal English birth cohort, we found that children living in lower-quality housing at age 7—particularly in damp and overcrowded homes—missed 15.5 more days during compulsory schooling. Housing quality was also associated with lower educational attainment; children living in overcrowded homes achieved lower average test scores in primary and secondary school. Given the importance of indoor environments for developing children’s lives, future research should consider implementing home environment checklists and/or more objective measurements (eg, sensors) in longitudinal cohort studies, explore lifelong health consequences of housing quality, as well as tracking the health and educational impacts of housing improvement policies over time.

## Supplementary material

10.1136/jech-2025-224495 online supplemental file 1

## Data Availability

Data are available in a public, open access repository.
