# Supplementary material for: Housing quality and school outcomes in England: a nationally representative linked cohort study
Source: J Epidemiol Community Health. 2025 Dec 16;80(4):e224495. doi: 10.1136/jech-2025-224495 (PMC13018768; doi:10.1136/jech-2025-224495)
Supplement: online supplemental file 1 [file jech-80-4-s001.docx]

**Supplementary Materials**

**Supplementary Figure S1**: Directed acyclic graph.

**Supplementary Figure S2**: Distribution of outcome variables - (A) percentage of missed session, and (B) and standardised test scores.

**Supplementary Figure S3**: Flowchart indicating sample selection.

**Supplementary Table S1**: Frequencies of housing condition features in the two analytical samples.

**Supplementary Figure S4:** Correlation coefficients (Kendall’s tau) between housing condition in the (A) Absence, and in the (B) Attainment samples

**Supplementary Table S2**: Number and percentage of total sessions missed across Year 1 to 11.

**Supplementary Table S3**: Association between each 1 SD decrease in housing quality and percentage of missed sessions in years 1 to 11.

**Supplementary Table S4**: Associations between ‘High versus ‘Low’ housing quality and percentage of missed sessions in years 1 to 11.

**Supplementary Table S5**: Associations between housing conditions and percentage of total missed sessions in years 1 to 11.

**Supplementary Table S6**: Associations between housing conditions and % of missed sessions in years 1 to 11 due to authorised absences.

**Supplementary Table S7**: Associations between housing conditions and % of missed sessions in years 1 to 11 due to unauthorised absences.

**Supplementary Table S8**: Average education attainment by housing quality.

**Supplementary Table S9**: Associations between ‘High’ versus ‘Low’ housing quality and educational attainment.

**Supplementary Table S10**: Associations between housing conditions and educational attainment.

**Supplementary Table S11**: Interaction between housing quality, household income/sex and % of missed sessions in years 1 to 11.

**Supplementary Table S12**: Interaction between housing quality, household income/sex and educational attainment.

**Supplementary Table S13**: Association between each 1 SD decrease in housing quality and percentage of missed sessions in years 1 to 11 among non-movers.

**Supplementary Table S14**: Association between each 1 SD decrease in housing quality and educational attainment among non-movers.

**Supplementary Table S15**: Association between each 1 SD decrease in housing quality and percentage of total missed sessions in years 1 to 11 after multiple imputation.

**Supplementary Table S16:** Association between each 1 SD decrease in housing quality and school performance after multiple imputation.

**Supplementary Table S17**: Associations between standardised housing conditions and percentage of total missed sessions in years 1 to 11.

**Supplementary Table S18**: Associations between standardised housing conditions and educational attainment.

**Supplementary Table S19**: Associations between housing quality scale and log-transformed percentage of total missed sessions in years 1 to 11.

**Supplementary Table S20**: Associations between housing quality items and log-transformed percentage of total missed sessions in years 1 to 11.

**Supplementary Table S21**: Associations between housing condition values and percentage of total missed sessions in years 1 to 11.

**Supplementary Table S22**: Associations between housing condition values and educational attainment.

**Supplementary Table S23**: Associations between housing quality scale, housing conditions and percentage of missed sessions across compulsory education using two methods of pooling across years.

**Supplementary Figure S1**: Directed acyclic graph. Note: housing quality is measured as a composite index of accommodation type, floor level, garden access, damp, heating, and overcrowding.


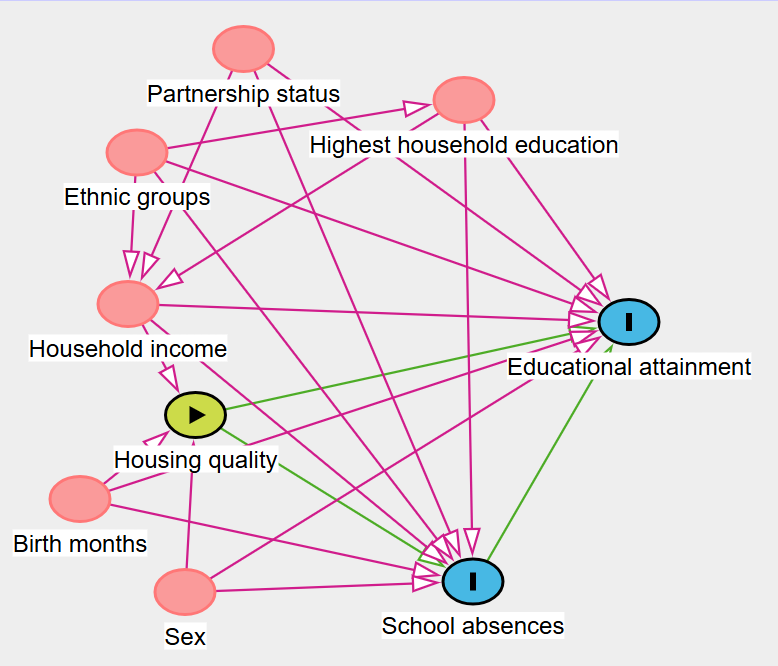


**Supplementary Figure S2:** Distribution of outcome variables - (A) percentage of missed session (n=7272), and (B) and standardised test scores (n=6741).

**(A)**

**
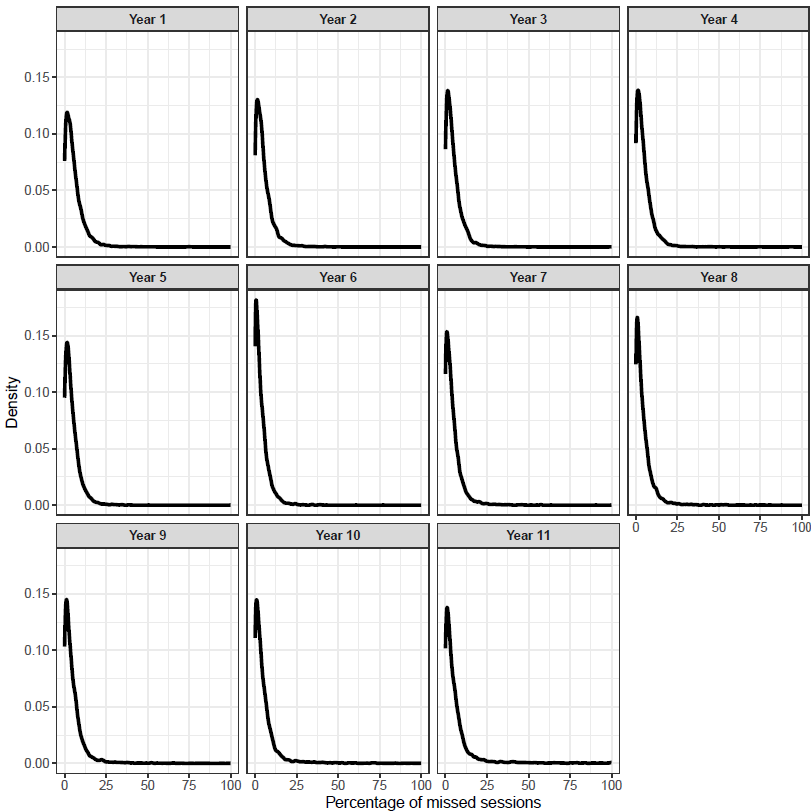
**

**(B)**

**
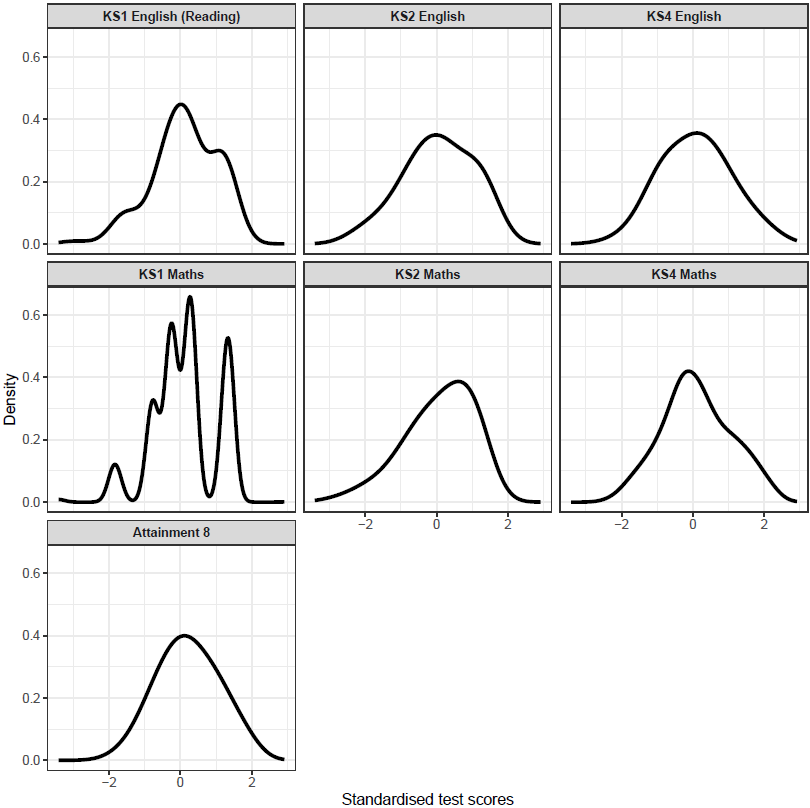
**

**Supplementary Figure S3:** Flowchart indicating sample selection in the Millennium Cohort Study

**
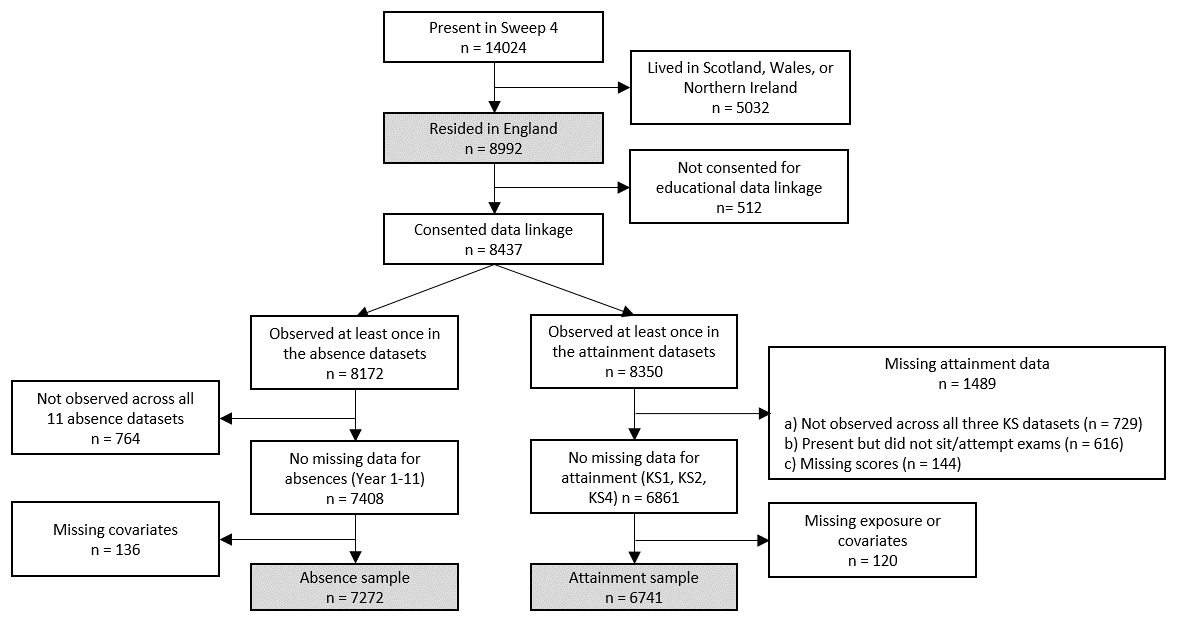
**

**Supplementary Table S1:** Frequencies of housing condition features in the two analytical samples of the Millennium Cohort Study.

|  | **Absence sample**  (n=7272) | **Attainment sample**  (n=6741) |
| --- | --- | --- |
| Type of accommodation |  |  |
| 0 (high) | 6651 (91.4%) | 6166 (91.5%) |
| 1 | 610 (8.4%) | 564 (8.3%) |
| 2 (low) | 11 (0.2%) | 11 (0.2%) |
| Floor level |  |  |
| 0 (high) | 6779 (93.3%) | 6285 (93.4%) |
| 1 | 238 (3.3%) | 216 (3.3%) |
| 2 (low) | 255 (3.3%) | 240 (3.3%) |
| Garden access |  |  |
| 0 (high) | 6538 (89.9%) | 6082 (90.4%) |
| 1 | 129 (2.0%) | 122 (2.0%) |
| 2 (low) | 605 (8.1%) | 537 (7.6%) |
| Damp and mould |  |  |
| 0 (high) | 6126 (84.3%) | 5724 (85.1%) |
| 1 | 956 (12.9%) | 856 (12.3%) |
| 2 (low) | 190 (2.8%) | 161 (2.6%) |
| Heating |  |  |
| 0 (high) | 6974 (95.5%) | 6476 (95.8%) |
| 1 | NA | NA |
| 2 (low) | 298 (4.5%) | 265 (4.2%) |
| Overcrowding |  |  |
| 0 (high) | 1075 (15.6%) | 1026 (16.1%) |
| 1 | 5173 (72.2%) | 4808 (72.4%) |
| 2 (low) | 1024 (12.3%) | 907 (11.6%) |

**Supplementary Figure S4:** Correlation coefficients (Kendall’s tau) between housing condition in the (A) Absence (n=7272), and in the (B) Attainment (n=6741) samples.

**
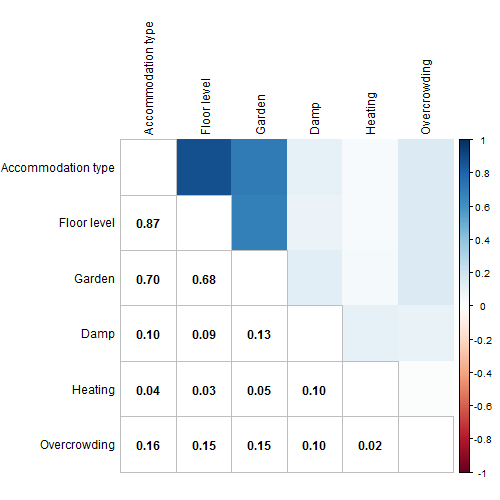
**


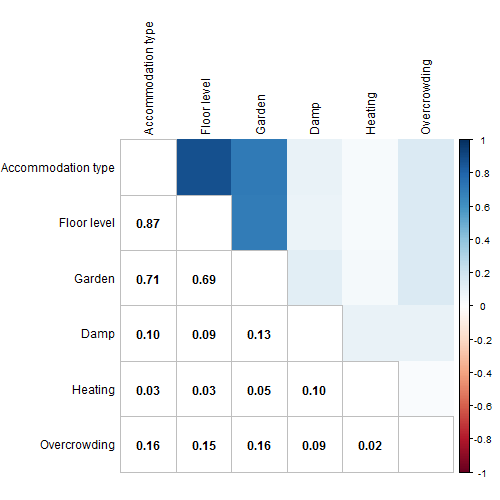


**Supplementary Table S2:** Number and percentage of total sessions missed across Year 1 to 11 in the Millennium Cohort Study.

| **Year** | **Total number of sessions missed** | | **Percentage of sessions missed** | |
| --- | --- | --- | --- | --- |
|  | Average | 95% CI | Average | 95% CI |
| Year 1 (2006/2007) | 16.98 | 16.39, 17.57 | 5.51 | 5.32, 5.71 |
| Year 2 (2007/2008) | 15.47 | 14.97, 15.98 | 5.07 | 4.90, 5.24 |
| Year 3 (2008/2009) | 14.66 | 14.18, 15.13 | 4.82 | 4.65, 4.98 |
| Year 4 (2009/2010) | 14.87 | 14.44, 15.31 | 4.84 | 4.70, 4.98 |
| Year 5 (2010/2011) | 13.97 | 13.57, 14.37 | 4.61 | 4.47, 4.74 |
| Year 6 (2011/2012) | 12.64 | 12.22, 13.07 | 4.01 | 3.88, 4.15 |
| Year 7 (2012/2013) | 14.17 | 13.64, 14.70 | 4.70 | 4.52, 4.87 |
| Year 8 (2013/2014) | 14.04 | 13.55, 14.54 | 4.67 | 4.50, 4.85 |
| Year 9 (2014/2015) | 16.61 | 15.98, 17.24 | 5.45 | 5.24, 5.66 |
| Year 10 (2015/2016) | 18.10 | 17.36, 18.83 | 5.83 | 5.59, 6.08 |
| Year 11 (2016/2017) | 20.86 | 19.83, 21.89 | 6.95 | 6.61, 7.30 |
| **Total (Year 1-11)** | **172.38** | **167.78, 176.98** | **5.13** | **4.99, 5.27** |
|  |  |  |  |  |
| **By housing quality** |  |  |  |  |
| High | 163.5 | 158.8, 168.1 | 4.86 | 4.72, 5.00 |
| Low | 216.9 | 204.2, 229.5 | 6.49 | 6.11, 6.88 |

Note: missed sessions were recorded by the Department for Education during the first 5 half terms. The sample size was n=7272.

**Supplementary Table S3:** Association between each 1 SD decrease in housing quality and percentage of missed sessions in years 1 to 11 in the Millennium Cohort Study.

|  | **Unadjusted** | | |  | **Adjusted** | | |
| --- | --- | --- | --- | --- | --- | --- | --- |
|  | b | 95% CI | *p* |  | b | 95% CI | *p* |
| Authorised absences |  |  |  |  |  |  |  |
| Year 1 | 0.47 | 0.35, 0.58 | <0.001 |  | 0.10 | -0.03, 0.23 | 0.122 |
| Year 2 | 0.45 | 0.34, 0.56 | <0.001 |  | 0.15 | 0.03, 0.26 | 0.012 |
| Year 3 | 0.43 | 0.31, 0.56 | <0.001 |  | 0.23 | 0.09, 0.36 | 0.001 |
| Year 4 | 0.25 | 0.13, 0.37 | <0.001 |  | 0.07 | -0.05, 0.19 | 0.230 |
| Year 5 | 0.26 | 0.15, 0.38 | <0.001 |  | 0.11 | -0.02, 0.24 | 0.085 |
| Year 6 | 0.21 | 0.12, 0.30 | <0.001 |  | 0.14 | 0.04, 0.23 | 0.004 |
| Year 7 | 0.29 | 0.16, 0.41 | <0.001 |  | 0.14 | 0.01, 0.27 | 0.029 |
| Year 8 | 0.22 | 0.10, 0.35 | 0.001 |  | 0.10 | -0.03, 0.24 | 0.140 |
| Year 9 | 0.23 | 0.11, 0.36 | <0.001 |  | 0.15 | 0.02, 0.28 | 0.025 |
| Year 10 | 0.20 | 0.05, 0.35 | 0.008 |  | 0.06 | -0.10, 0.22 | 0.450 |
| Year 11 | 0.27 | 0.02, 0.52 | 0.035 |  | 0.12 | -0.09, 0.32 | 0.259 |
| Total | 0.30 | 0.21, 0.38 | <0.001 |  | 0.12 | 0.04, 0.21 | 0.005 |
| Unauthorised absences |  |  |  |  |  |  |  |
| Year 1 | 0.26 | 0.19, 0.34 | <0.001 |  | 0.13 | 0.05, 0.21 | 0.001 |
| Year 2 | 0.22 | 0.15, 0.28 | <0.001 |  | 0.09 | 0.02, 0.15 | 0.012 |
| Year 3 | 0.22 | 0.14, 0.30 | <0.001 |  | 0.08 | 0.01, 0.15 | 0.029 |
| Year 4 | 0.24 | 0.16, 0.33 | <0.001 |  | 0.12 | 0.04, 0.20 | 0.005 |
| Year 5 | 0.18 | 0.12, 0.25 | <0.001 |  | 0.06 | -0.01, 0.13 | 0.093 |
| Year 6 | 0.15 | 0.10, 0.20 | <0.001 |  | 0.02 | -0.03, 0.08 | 0.435 |
| Year 7 | 0.20 | 0.12, 0.28 | <0.001 |  | 0.06 | -0.02, 0.14 | 0.129 |
| Year 8 | 0.24 | 0.14, 0.34 | <0.001 |  | 0.05 | -0.06, 0.17 | 0.337 |
| Year 9 | 0.44 | 0.29, 0.59 | <0.001 |  | 0.17 | 0.01, 0.33 | 0.034 |
| Year 10 | 0.61 | 0.41, 0.82 | <0.001 |  | 0.24 | 0.02, 0.47 | 0.032 |
| Year 11 | 0.79 | 0.49, 1.09 | <0.001 |  | 0.28 | -0.02, 0.58 | 0.066 |
| Total | 0.32 | 0.26, 0.39 | <0.001 |  | 0.12 | 0.05, 0.19 | 0.001 |
| Total absences |  |  |  |  |  |  |  |
| Year 1 | 0.73 | 0.58, 0.88 | <0.001 |  | 0.23 | 0.07, 0.40 | 0.006 |
| Year 2 | 0.67 | 0.54, 0.80 | <0.001 |  | 0.23 | 0.09, 0.37 | 0.001 |
| Year 3 | 0.65 | 0.51, 0.79 | <0.001 |  | 0.31 | 0.16, 0.46 | <0.001 |
| Year 4 | 0.50 | 0.36, 0.63 | <0.001 |  | 0.19 | 0.05, 0.34 | 0.009 |
| Year 5 | 0.45 | 0.32, 0.58 | <0.001 |  | 0.17 | 0.03, 0.32 | 0.020 |
| Year 6 | 0.36 | 0.25, 0.47 | <0.001 |  | 0.16 | 0.05, 0.28 | 0.007 |
| Year 7 | 0.49 | 0.32, 0.66 | <0.001 |  | 0.21 | 0.03, 0.39 | 0.023 |
| Year 8 | 0.46 | 0.27, 0.65 | <0.001 |  | 0.16 | -0.04, 0.36 | 0.125 |
| Year 9 | 0.67 | 0.45, 0.90 | <0.001 |  | 0.32 | 0.09, 0.56 | 0.008 |
| Year 10 | 0.82 | 0.51, 1.12 | <0.001 |  | 0.30 | -0.02, 0.62 | 0.064 |
| Year 11 | 1.06 | 0.62, 1.49 | <0.001 |  | 0.40 | 0.00, 0.79 | 0.050 |
| Total | 0.62 | 0.50, 0.75 | <0.001 |  | 0.24 | 0.10, 0.38 | 0.001 |

Linear regressions with complex survey weights were conducted separately. Adjusted models controlled for sex, month of birth, ethnic groups, maternal partnership status, highest household education, and household income. The sample size was n=7272.

**Supplementary Table S4:** Associations between *‘High* versus *‘Low’* housing quality and percentage of missed sessions in years 1 to 11 in the Millennium Cohort Study.

|  | **Unadjusted** | | |  | **Adjusted** | | |
| --- | --- | --- | --- | --- | --- | --- | --- |
|  | b | 95% CI | *p* |  | b | 95% CI | *p* |
| Total absences |  |  |  |  |  |  |  |
| Year 1 | 2.03 | 1.59, 2.47 | <0.001 |  | 0.94 | 0.50, 1.38 | <0.001 |
| Year 2 | 1.80 | 1.42, 2.18 | <0.001 |  | 0.84 | 0.44, 1.24 | <0.001 |
| Year 3 | 1.75 | 1.34, 2.15 | <0.001 |  | 0.97 | 0.57, 1.37 | <0.001 |
| Year 4 | 1.23 | 0.85, 1.61 | <0.001 |  | 0.53 | 0.14, 0.91 | 0.007 |
| Year 5 | 1.12 | 0.73, 1.50 | <0.001 |  | 0.47 | 0.07, 0.87 | 0.022 |
| Year 6 | 0.94 | 0.62, 1.26 | <0.001 |  | 0.46 | 0.09, 0.83 | 0.016 |
| Year 7 | 1.24 | 0.80, 1.68 | <0.001 |  | 0.55 | 0.11, 1.00 | 0.016 |
| Year 8 | 1.19 | 0.67, 1.71 | <0.001 |  | 0.45 | -0.07, 0.97 | 0.092 |
| Year 9 | 1.88 | 1.18, 2.58 | <0.001 |  | 1.03 | 0.33, 1.72 | 0.004 |
| Year 10 | 2.08 | 1.27, 2.90 | <0.001 |  | 0.85 | 0.03, 1.66 | 0.042 |
| Year 11 | 2.66 | 1.55, 3.78 | <0.001 |  | 1.04 | 0.03, 2.06 | 0.043 |
| Total | 1.63 | 1.22, 2.04 | <0.001 |  | 0.74 | 0.34, 1.13 | <0.001 |

Linear regressions with complex survey weights were conducted separately. Adjusted models controlled for sex, month of birth, ethnic groups, maternal partnership status, highest household education, and household income. The sample size was n=7272.

**Supplementary Table S5:** Associations between housing conditions and percentage of total missed sessions in years 1 to 11 in the Millennium Cohort Study.

|  | **Adjusted** | | | | | | |
| --- | --- | --- | --- | --- | --- | --- | --- |
|  | b | 95% CI | *p* |  | b | 95% CI | *p* |
|  | Accommodation type | | |  | Floor level | | |
| Year 1 | 0.54 | 0.02, 1.06 | 0.041 |  | 0.23 | -0.22, 0.68 | 0.308 |
| Year 2 | 0.57 | 0.06, 1.07 | 0.029 |  | 0.33 | -0.08, 0.75 | 0.115 |
| Year 3 | 0.64 | 0.10, 1.18 | 0.020 |  | 0.28 | -0.13, 0.69 | 0.180 |
| Year 4 | 0.67 | 0.23, 1.11 | 0.003 |  | 0.17 | -0.20, 0.54 | 0.358 |
| Year 5 | 0.82 | 0.30, 1.34 | 0.002 |  | 0.34 | -0.09, 0.77 | 0.117 |
| Year 6 | 0.63 | 0.14, 1.11 | 0.012 |  | 0.16 | -0.16, 0.48 | 0.325 |
| Year 7 | 0.33 | -0.26, 0.93 | 0.273 |  | 0.23 | -0.27, 0.73 | 0.370 |
| Year 8 | 0.29 | -0.40, 0.98 | 0.405 |  | 0.17 | -0.41, 0.75 | 0.564 |
| Year 9 | 0.44 | -0.44, 1.32 | 0.328 |  | 0.29 | -0.37, 0.94 | 0.394 |
| Year 10 | 0.44 | -0.59, 1.47 | 0.400 |  | 0.45 | -0.47, 1.37 | 0.336 |
| Year 11 | 0.45 | -0.89, 1.79 | 0.509 |  | 0.61 | -0.56, 1.78 | 0.306 |
| Total | **0.53** | **0.03, 1.02** | **0.037** |  | 0.30 | -0.13, 0.72 | 0.170 |
|  | Garden access | | |  | Damp | | |
| Year 1 | 0.07 | -0.19, 0.33 | 0.601 |  | 0.53 | 0.17, 0.90 | 0.004 |
| Year 2 | 0.16 | -0.09, 0.41 | 0.203 |  | 0.32 | 0.05, 0.59 | 0.020 |
| Year 3 | 0.44 | 0.13, 0.75 | 0.006 |  | 0.52 | 0.25, 0.79 | <0.001 |
| Year 4 | 0.19 | -0.08, 0.46 | 0.161 |  | 0.34 | 0.04, 0.64 | 0.025 |
| Year 5 | 0.15 | -0.12, 0.42 | 0.277 |  | 0.12 | -0.13, 0.37 | 0.329 |
| Year 6 | 0.12 | -0.12, 0.36 | 0.315 |  | 0.20 | -0.06, 0.47 | 0.127 |
| Year 7 | 0.17 | -0.17, 0.51 | 0.321 |  | 0.40 | 0.08, 0.71 | 0.013 |
| Year 8 | 0.16 | -0.21, 0.54 | 0.387 |  | 0.23 | -0.13, 0.58 | 0.209 |
| Year 9 | 0.27 | -0.22, 0.76 | 0.277 |  | 0.75 | 0.30, 1.19 | 0.001 |
| Year 10 | 0.33 | -0.19, 0.86 | 0.215 |  | 0.95 | 0.39, 1.50 | 0.001 |
| Year 11 | 0.55 | -0.16, 1.26 | 0.129 |  | 0.87 | 0.19, 1.54 | 0.012 |
| Total | 0.24 | -0.02, 0.5 | 0.076 |  | **0.47** | **0.24, 0.71** | **<0.001** |
|  | Heating | | |  | Overcrowding | | |
| Year 1 | 0.36 | 0.07, 0.66 | 0.015 |  | 0.21 | -0.05, 0.48 | 0.109 |
| Year 2 | 0.31 | -0.03, 0.65 | 0.070 |  | 0.24 | 0.00, 0.49 | 0.050 |
| Year 3 | 0.08 | -0.22, 0.37 | 0.618 |  | 0.36 | 0.11, 0.61 | 0.005 |
| Year 4 | 0.04 | -0.30, 0.38 | 0.820 |  | 0.23 | -0.03, 0.49 | 0.084 |
| Year 5 | 0.07 | -0.22, 0.36 | 0.629 |  | 0.18 | -0.04, 0.40 | 0.115 |
| Year 6 | 0.04 | -0.25, 0.34 | 0.774 |  | 0.27 | 0.07, 0.48 | 0.010 |
| Year 7 | -0.02 | -0.32, 0.28 | 0.889 |  | 0.41 | 0.11, 0.71 | 0.007 |
| Year 8 | 0.00 | -0.38, 0.38 | 0.993 |  | 0.31 | 0.05, 0.58 | 0.021 |
| Year 9 | -0.02 | -0.50, 0.45 | 0.924 |  | 0.59 | 0.22, 0.96 | 0.002 |
| Year 10 | -0.42 | -0.89, 0.05 | 0.078 |  | 0.42 | -0.14, 0.97 | 0.142 |
| Year 11 | -0.42 | -1.11, 0.26 | 0.226 |  | 0.66 | 0.07, 1.24 | 0.027 |
| Total | 0.00 | -0.26, 0.26 | 0.990 |  | **0.35** | **0.13, 0.58** | **0.002** |

Linear regressions with complex survey weights were conducted separately. Adjusted models controlled for sex, month of birth, ethnic groups, maternal partnership status, highest household education, and household income. The sample size was n=7272.

**Supplementary Table S6:** Associations between housing conditions and % of missed sessions in years 1 to 11 due to authorised absences in the Millennium Cohort Study.

|  | **Adjusted** | | | | | | |
| --- | --- | --- | --- | --- | --- | --- | --- |
|  | b | 95% CI | *p* |  | b | 95% CI | *p* |
|  | Accommodation type | | |  | Floor level | | |
| Year 1 | 0.17 | -0.26, 0.60 | 0.436 |  | -0.02 | -0.35, 0.31 | 0.913 |
| Year 2 | 0.36 | -0.05, 0.77 | 0.085 |  | 0.20 | -0.13, 0.54 | 0.239 |
| Year 3 | 0.51 | 0.06, 0.96 | 0.025 |  | 0.18 | -0.17, 0.52 | 0.313 |
| Year 4 | 0.35 | -0.03, 0.73 | 0.072 |  | 0.08 | -0.25, 0.41 | 0.637 |
| Year 5 | 0.56 | 0.06, 1.07 | 0.029 |  | 0.22 | -0.17, 0.61 | 0.274 |
| Year 6 | 0.61 | 0.18, 1.03 | 0.005 |  | 0.20 | -0.07, 0.47 | 0.140 |
| Year 7 | 0.32 | -0.14, 0.79 | 0.171 |  | 0.15 | -0.20, 0.49 | 0.398 |
| Year 8 | 0.26 | -0.24, 0.77 | 0.305 |  | 0.11 | -0.28, 0.50 | 0.572 |
| Year 9 | 0.12 | -0.31, 0.55 | 0.576 |  | 0.04 | -0.25, 0.33 | 0.789 |
| Year 10 | 0.20 | -0.31, 0.70 | 0.445 |  | 0.06 | -0.33, 0.44 | 0.777 |
| Year 11 | 0.01 | -0.57, 0.60 | 0.967 |  | 0.01 | -0.58, 0.61 | 0.964 |
| Total | **0.32** | **0.00, 0.63** | **0.048** |  | 0.11 | -0.14, 0.37 | 0.388 |
|  | Garden access | | |  | Damp and mould | | |
| Year 1 | -0.06 | -0.27, 0.15 | 0.565 |  | 0.26 | -0.01, 0.54 | 0.060 |
| Year 2 | 0.06 | -0.13, 0.25 | 0.533 |  | 0.28 | 0.03, 0.52 | 0.026 |
| Year 3 | 0.20 | -0.04, 0.44 | 0.094 |  | 0.55 | 0.32, 0.78 | <0.001 |
| Year 4 | 0.00 | -0.21, 0.21 | 0.990 |  | 0.28 | 0.02, 0.55 | 0.035 |
| Year 5 | 0.03 | -0.19, 0.26 | 0.767 |  | 0.18 | -0.04, 0.39 | 0.117 |
| Year 6 | 0.11 | -0.07, 0.29 | 0.210 |  | 0.18 | -0.04, 0.40 | 0.115 |
| Year 7 | 0.06 | -0.18, 0.31 | 0.613 |  | 0.39 | 0.13, 0.64 | 0.004 |
| Year 8 | 0.04 | -0.24, 0.31 | 0.798 |  | 0.23 | 0.00, 0.45 | 0.045 |
| Year 9 | -0.06 | -0.30, 0.18 | 0.609 |  | 0.55 | 0.26, 0.85 | <0.001 |
| Year 10 | 0.04 | -0.22, 0.29 | 0.774 |  | 0.34 | 0.06, 0.63 | 0.017 |
| Year 11 | 0.00 | -0.31, 0.30 | 0.982 |  | 0.41 | 0.00, 0.82 | 0.053 |
| Total | 0.04 | -0.12, 0.20 | 0.634 |  | **0.33** | **0.18, 0.48** | **<0.001** |
|  | Heating | | |  | Overcrowding | | |
| Year 1 | 0.35 | 0.09, 0.62 | 0.009 |  | 0.12 | -0.12, 0.37 | 0.310 |
| Year 2 | 0.25 | -0.04, 0.55 | 0.094 |  | 0.10 | -0.12, 0.33 | 0.361 |
| Year 3 | 0.14 | -0.11, 0.39 | 0.264 |  | 0.21 | 0.01, 0.42 | 0.043 |
| Year 4 | -0.03 | -0.30, 0.25 | 0.837 |  | 0.03 | -0.17, 0.24 | 0.741 |
| Year 5 | 0.09 | -0.14, 0.33 | 0.428 |  | 0.06 | -0.12, 0.25 | 0.503 |
| Year 6 | 0.09 | -0.18, 0.36 | 0.517 |  | 0.12 | -0.06, 0.30 | 0.194 |
| Year 7 | 0.01 | -0.20, 0.23 | 0.892 |  | 0.21 | -0.01, 0.44 | 0.066 |
| Year 8 | 0.01 | -0.24, 0.26 | 0.941 |  | 0.19 | -0.01, 0.40 | 0.062 |
| Year 9 | 0.10 | -0.25, 0.44 | 0.577 |  | 0.33 | 0.07, 0.59 | 0.014 |
| Year 10 | -0.11 | -0.42, 0.20 | 0.483 |  | -0.01 | -0.35, 0.32 | 0.934 |
| Year 11 | 0.00 | -0.41, 0.42 | 0.991 |  | 0.30 | -0.09, 0.69 | 0.134 |
| Total | 0.08 | -0.10, 0.27 | 0.374 |  | 0.15 | -0.01, 0.32 | 0.066 |

Linear regressions with complex survey weights were conducted separately. Adjusted models controlled for sex, month of birth, ethnic groups, maternal partnership status, highest household education, and household income. The sample size was n=7272.

**Supplementary Table S7:** Associations between housing conditions and % of missed sessions in years 1 to 11 due to unauthorised absences in the Millennium Cohort Study.

|  | **Adjusted** | | | | | | |
| --- | --- | --- | --- | --- | --- | --- | --- |
|  | b | 95% CI | *p* |  | b | 95% CI | *p* |
|  | Accommodation type | | |  | Floor level | | |
| Year 1 | 0.37 | 0.13, 0.61 | 0.003 |  | 0.25 | 0.04, 0.46 | 0.021 |
| Year 2 | 0.21 | -0.02, 0.44 | 0.077 |  | 0.13 | -0.05, 0.32 | 0.160 |
| Year 3 | 0.13 | -0.16, 0.42 | 0.376 |  | 0.10 | -0.10, 0.31 | 0.311 |
| Year 4 | 0.32 | 0.06, 0.58 | 0.015 |  | 0.10 | -0.06, 0.25 | 0.217 |
| Year 5 | 0.26 | 0.01, 0.51 | 0.042 |  | 0.12 | 0.00, 0.25 | 0.055 |
| Year 6 | 0.02 | -0.20, 0.23 | 0.874 |  | -0.04 | -0.15, 0.07 | 0.487 |
| Year 7 | 0.01 | -0.29, 0.30 | 0.954 |  | 0.08 | -0.17, 0.32 | 0.528 |
| Year 8 | 0.03 | -0.33, 0.39 | 0.880 |  | 0.06 | -0.26, 0.38 | 0.713 |
| Year 9 | 0.32 | -0.31, 0.94 | 0.323 |  | 0.25 | -0.22, 0.71 | 0.299 |
| Year 10 | 0.24 | -0.42, 0.91 | 0.474 |  | 0.39 | -0.24, 1.03 | 0.225 |
| Year 11 | 0.44 | -0.61, 1.49 | 0.407 |  | 0.60 | -0.33, 1.53 | 0.208 |
| Total | 0.21 | -0.07, 0.49 | 0.132 |  | 0.19 | -0.05, 0.42 | 0.124 |
|  | Garden access | | |  | Damp and mould | | |
| Year 1 | 0.13 | 0.01, 0.25 | 0.031 |  | 0.27 | 0.07, 0.47 | 0.008 |
| Year 2 | 0.10 | -0.05, 0.25 | 0.175 |  | 0.04 | -0.06, 0.15 | 0.433 |
| Year 3 | 0.24 | 0.01, 0.47 | 0.045 |  | -0.03 | -0.13, 0.07 | 0.586 |
| Year 4 | 0.20 | 0.00, 0.39 | 0.050 |  | 0.06 | -0.06, 0.18 | 0.350 |
| Year 5 | 0.12 | -0.05, 0.28 | 0.179 |  | -0.05 | -0.17, 0.07 | 0.381 |
| Year 6 | 0.01 | -0.13, 0.14 | 0.929 |  | 0.03 | -0.09, 0.15 | 0.670 |
| Year 7 | 0.11 | -0.05, 0.26 | 0.179 |  | 0.01 | -0.15, 0.17 | 0.894 |
| Year 8 | 0.13 | -0.07, 0.33 | 0.209 |  | 0.00 | -0.22, 0.22 | 0.982 |
| Year 9 | 0.33 | -0.01, 0.68 | 0.057 |  | 0.19 | -0.09, 0.48 | 0.188 |
| Year 10 | 0.29 | -0.08, 0.67 | 0.124 |  | 0.60 | 0.19, 1.02 | 0.005 |
| Year 11 | 0.55 | -0.02, 1.13 | 0.058 |  | 0.46 | -0.06, 0.99 | 0.083 |
| Total | **0.20** | **0.03, 0.37** | **0.020** |  | **0.14** | **0.00, 0.28** | **0.046** |
|  | Heating | | |  | Overcrowding | | |
| Year 1 | 0.01 | -0.10, 0.12 | 0.837 |  | 0.09 | -0.02, 0.19 | 0.095 |
| Year 2 | 0.06 | -0.07, 0.19 | 0.379 |  | 0.14 | 0.04, 0.25 | 0.008 |
| Year 3 | -0.07 | -0.16, 0.03 | 0.178 |  | 0.15 | -0.01, 0.30 | 0.064 |
| Year 4 | 0.07 | -0.11, 0.25 | 0.450 |  | 0.19 | 0.04, 0.35 | 0.015 |
| Year 5 | -0.03 | -0.16, 0.11 | 0.696 |  | 0.12 | -0.03, 0.26 | 0.107 |
| Year 6 | -0.05 | -0.16, 0.06 | 0.395 |  | 0.15 | 0.03, 0.27 | 0.012 |
| Year 7 | -0.04 | -0.19, 0.12 | 0.659 |  | 0.20 | 0.05, 0.34 | 0.008 |
| Year 8 | -0.01 | -0.28, 0.27 | 0.955 |  | 0.12 | -0.05, 0.30 | 0.172 |
| Year 9 | -0.12 | -0.42, 0.19 | 0.442 |  | 0.26 | 0.01, 0.51 | 0.042 |
| Year 10 | -0.31 | -0.59, -0.03 | 0.030 |  | 0.43 | 0.05, 0.81 | 0.025 |
| Year 11 | -0.42 | -0.80, -0.05 | 0.026 |  | 0.36 | -0.05, 0.76 | 0.085 |
| Total | -0.08 | -0.21, 0.05 | 0.225 |  | **0.20** | **0.07, 0.33** | **0.003** |

Linear regressions with complex survey weights were conducted separately. Adjusted models controlled for sex, month of birth, ethnic groups, maternal partnership status, highest household education, and household income. The sample size was n=7272.

**Supplementary Table S8:** Average education attainment by housing quality in the Millennium Cohort Study.

|  | **Higher housing quality** | |  | **Lower housing quality** | |
| --- | --- | --- | --- | --- | --- |
|  | Mean | 95% CI |  | Mean | 95% CI |
| KS1 - English (Reading) | 0.16 | 0.14, 0.18 |  | -0.11 | -0.16, -0.05 |
| KS2 - English | 0.08 | 0.05, 0.10 |  | -0.16 | -0.22, -0.11 |
| KS4 - English | 0.11 | 0.09, 0.14 |  | -0.13 | -0.19, -0.07 |
| KS1 - Math | 0.16 | 0.13, 0.18 |  | -0.11 | -0.17, -0.06 |
| KS2 - Math | 0.10 | 0.07, 0.12 |  | -0.14 | -0.20, -0.08 |
| KS4 - Math | 0.15 | 0.12, 0.17 |  | -0.12 | -0.18, -0.06 |
| KS4 - Attainment 8 | 0.26 | 0.24, 0.28 |  | 0.00 | -0.05, 0.05 |

The sample size was n=6741.

**Supplementary Table S9**: Associations between *‘Higher’* versus *‘Lower’* housing quality and educational attainment in the Millennium Cohort Study.

|  | **Unadjusted** | | |  | **Adjusted** | | |
| --- | --- | --- | --- | --- | --- | --- | --- |
|  | β | 95% CI | *p* |  | β | 95% CI | *p* |
| Key Stage 1 |  |  |  |  |  |  |  |
| English (Reading) | -0.31 | -0.37, -0.24 | <0.001 |  | -0.07 | -0.13, -0.01 | 0.033 |
| Math | -0.29 | -0.36, -0.22 | <0.001 |  | -0.08 | -0.15, -0.01 | 0.019 |
| Key Stage 2 |  |  |  |  |  |  |  |
| English | -0.28 | -0.35, -0.21 | <0.001 |  | -0.06 | -0.12, 0.01 | 0.093 |
| Math | -0.30 | -0.37, -0.22 | <0.001 |  | -0.12 | -0.2, -0.04 | 0.002 |
| Key Stage 4 |  |  |  |  |  |  |  |
| English | -0.34 | -0.42, -0.27 | <0.001 |  | -0.12 | -0.19, -0.05 | 0.001 |
| Math | -0.35 | -0.42, -0.27 | <0.001 |  | -0.11 | -0.18, -0.03 | 0.004 |
| Attainment 8 | -0.35 | -0.42, -0.28 | <0.001 |  | -0.13 | -0.19, -0.07 | <0.001 |

Linear regressions with complex survey weights were conducted. Adjusted models controlled for sex, month of birth, ethnic groups, maternal partnership status, highest household education, and household income. The sample size was n=6741.

**Supplementary Table S10:** Associations between housing conditions and educational attainment in the Millennium Cohort Study.

|  | **Adjusted** | | | | | | |
| --- | --- | --- | --- | --- | --- | --- | --- |
|  | b | 95% CI | *p* |  | b | 95% CI | *p* |
|  | Accommodation type | | |  | Floor level | | |
| KS1: English (Reading) | -0.03 | -0.12, 0.06 | 0.553 |  | 0.01 | -0.04, 0.07 | 0.687 |
| KS1: Math | -0.02 | -0.12, 0.07 | 0.656 |  | 0.01 | -0.04, 0.07 | 0.603 |
| KS2: English | 0.04 | -0.05, 0.12 | 0.396 |  | 0.04 | -0.02, 0.09 | 0.208 |
| KS2: Math | -0.08 | -0.17, 0.02 | 0.123 |  | -0.03 | -0.10, 0.03 | 0.345 |
| KS4: English | -0.01 | -0.11, 0.08 | 0.784 |  | -0.01 | -0.08, 0.06 | 0.823 |
| KS4: Math | -0.06 | -0.15, 0.04 | 0.253 |  | -0.02 | -0.09, 0.04 | 0.480 |
| KS4: Attainment 8 | -0.05 | -0.14, 0.03 | 0.223 |  | -0.03 | -0.09, 0.04 | 0.436 |
|  | Garden access | | |  | Damp | | |
| KS1: English (Reading) | -0.02 | -0.06, 0.03 | 0.439 |  | 0.01 | -0.04, 0.07 | 0.587 |
| KS1: Math | -0.02 | -0.06, 0.02 | 0.355 |  | 0.01 | -0.03, 0.06 | 0.559 |
| KS2: English | -0.01 | -0.06, 0.04 | 0.737 |  | 0.03 | -0.03, 0.08 | 0.347 |
| KS2: Math | -0.04 | -0.09, 0.01 | 0.114 |  | -0.01 | -0.06, 0.04 | 0.725 |
| KS4: English | -0.04 | -0.09, 0.01 | 0.115 |  | -0.01 | -0.06, 0.04 | 0.714 |
| KS4: Math | -0.02 | -0.07, 0.03 | 0.366 |  | -0.02 | -0.07, 0.03 | 0.346 |
| KS4: Attainment 8 | -0.03 | -0.07, 0.01 | 0.170 |  | -0.02 | -0.07, 0.02 | 0.326 |
|  | Heating | | |  | Overcrowding | | |
| KS1: English (Reading) | -0.04 | -0.10, 0.01 | 0.098 |  | -0.12 | -0.16, -0.07 | <0.001 |
| KS1: Math | -0.06 | -0.11, 0.00 | 0.045 |  | -0.10 | -0.14, -0.06 | <0.001 |
| KS2: English | -0.06 | -0.12, 0.00 | 0.067 |  | -0.15 | -0.19, -0.10 | <0.001 |
| KS2: Math | -0.07 | -0.13, 0.00 | 0.048 |  | -0.10 | -0.14, -0.05 | <0.001 |
| KS4: English | -0.05 | -0.10, 0.01 | 0.104 |  | -0.11 | -0.16, -0.06 | <0.001 |
| KS4: Math | -0.04 | -0.09, 0.01 | 0.154 |  | -0.09 | -0.14, -0.04 | <0.001 |
| KS4: Attainment 8 | -0.05 | -0.10, 0.00 | 0.066 |  | -0.12 | -0.16, -0.07 | <0.001 |

Linear regressions with complex survey weights were conducted separately. Adjusted models controlled for sex, month of birth, ethnic groups, maternal partnership status, highest household education, and household income. The sample size was n=6741.

**Supplementary Table S11:** Interaction between housing quality, household income/sex and % of missed sessions in years 1 to 11 in the Millennium Cohort Study.

|  | **Household Income** | | |  | **Sex** | | |
| --- | --- | --- | --- | --- | --- | --- | --- |
|  | F-value | *p* | *p_FDR_* |  | F-value | *p* | *p_FDR_* |
| Total absences |  |  |  |  |  |  |  |
| Year 1 | 0.679 | 0.608 | 0.678 |  | 1.060 | 0.304 | 0.544 |
| Year 2 | 1.356 | 0.250 | 0.678 |  | 0.003 | 0.957 | 0.970 |
| Year 3 | 0.741 | 0.565 | 0.678 |  | 0.001 | 0.970 | 0.970 |
| Year 4 | 1.055 | 0.380 | 0.678 |  | 0.725 | 0.395 | 0.544 |
| Year 5 | 3.705 | 0.006 | 0.068 |  | 2.428 | 0.121 | 0.544 |
| Year 6 | 0.848 | 0.496 | 0.678 |  | 0.191 | 0.663 | 0.810 |
| Year 7 | 0.553 | 0.697 | 0.697 |  | 0.798 | 0.373 | 0.544 |
| Year 8 | 1.500 | 0.203 | 0.678 |  | 1.720 | 0.191 | 0.544 |
| Year 9 | 0.926 | 0.450 | 0.678 |  | 2.259 | 0.134 | 0.544 |
| Year 10 | 0.666 | 0.616 | 0.678 |  | 1.330 | 0.250 | 0.544 |
| Year 11 | 0.764 | 0.550 | 0.678 |  | 1.057 | 0.305 | 0.544 |
| Total | 0.401 | 0.808 | NA |  | 0.809 | 0.369 | NA |

Linear regressions with complex survey weights were conducted separately. Models were controlled for sex, month of birth, ethnic groups, maternal partnership status, highest household education, and household income. The sample size was n=7272.

**Supplementary Table S12:** Interaction between housing quality, household income/sex and educational attainment in the Millennium Cohort Study.

|  | **Household Income** | |  | **Sex** | |
| --- | --- | --- | --- | --- | --- |
|  | F-value | *p* |  | F-value | *p* |
| KS1: English (Reading) | 1.966 | 0.101 |  | 0.215 | 0.643 |
| KS1: Math | 2.342 | 0.056 |  | 1.838 | 0.177 |
| KS2: English | 0.854 | 0.493 |  | 0.009 | 0.922 |
| KS2: Math | 1.608 | 0.174 |  | 0.943 | 0.333 |
| KS4: English | 1.447 | 0.220 |  | 0.165 | 0.685 |
| KS4: Math | 2.156 | 0.075 |  | 0.670 | 0.414 |
| KS4: Attainment 8 | 1.147 | 0.336 |  | 0.235 | 0.629 |

Linear regressions with complex survey weights were conducted separately. Models were controlled for sex, month of birth, ethnic groups, maternal partnership status, highest household education, and household income. The sample size was n=6741.

**Supplementary Table S13:** Association between each 1 SD decrease in housing quality and percentage of missed sessions in years 1 to 11 among non-movers in the Millennium Cohort Study.

|  | **Sample size** | **Unadjusted** | | |  | **Adjusted** | | |
| --- | --- | --- | --- | --- | --- | --- | --- | --- |
|  |  | b | 95% CI | *p* |  | b | 95% CI | *p* |
| Total absences |  |  |  |  |  |  |  |  |
| Year 1 | 6603 | 0.75 | 0.59, 0.90 | <0.001 |  | 0.26 | 0.09, 0.44 | 0.003 |
| Year 2 | 6603 | 0.66 | 0.53, 0.79 | <0.001 |  | 0.23 | 0.08, 0.37 | 0.003 |
| Year 3 | 7272 | 0.65 | 0.51, 0.79 | <0.001 |  | 0.31 | 0.16, 0.46 | <0.001 |
| Year 4 | 7272 | 0.50 | 0.36, 0.63 | <0.001 |  | 0.19 | 0.05, 0.34 | 0.009 |
| Year 5 | 7272 | 0.45 | 0.32, 0.58 | <0.001 |  | 0.17 | 0.03, 0.32 | 0.020 |
| Year 6 | 7272 | 0.36 | 0.25, 0.47 | <0.001 |  | 0.16 | 0.05, 0.28 | 0.007 |
| Year 7 | 5279 | 0.43 | 0.22, 0.65 | <0.001 |  | 0.16 | -0.06, 0.38 | 0.145 |
| Year 8 | 5279 | 0.24 | 0.09, 0.40 | 0.002 |  | -0.06 | -0.22, 0.11 | 0.492 |
| Year 9 | 5279 | 0.44 | 0.23, 0.65 | <0.001 |  | 0.11 | -0.11, 0.33 | 0.335 |
| Year 10 | 4091 | 0.68 | 0.33, 1.02 | <0.001 |  | 0.26 | -0.11, 0.64 | 0.167 |
| Year 11 | 4091 | 0.70 | 0.26, 1.14 | 0.002 |  | 0.23 | -0.22, 0.69 | 0.311 |
| Total | 66313 | 0.53 | 0.42, 0.64 | <0.001 |  | 0.19 | 0.07, 0.30 | 0.001 |

Linear regressions with complex survey weights were conducted separately. Adjusted models controlled for sex, month of birth, ethnic groups, maternal partnership status, highest household education, and household income.

**Supplementary Table S14**: Association between each 1 SD decrease in housing quality and educational attainment among non-movers in the Millennium Cohort Study.

|  | **Sample size** | **Unadjusted** | | |  | **Adjusted** | | |
| --- | --- | --- | --- | --- | --- | --- | --- | --- |
|  |  | β | 95% CI | *p* |  | β | 95% CI | *p* |
| Key Stage 1 |  |  |  |  |  |  |  |  |
| English (Reading) | 6138 | -0.14 | -0.16, -0.12 | <0.001 |  | -0.03 | -0.05, 0.00 | 0.019 |
| Math | 6138 | -0.12 | -0.15, -0.10 | <0.001 |  | -0.03 | -0.05, 0.00 | 0.042 |
| Key Stage 2 |  |  |  |  |  |  |  |  |
| English | 6741 | -0.12 | -0.15, -0.09 | <0.001 |  | -0.02 | -0.05, 0.00 | 0.060 |
| Math | 6741 | -0.12 | -0.15, -0.09 | <0.001 |  | -0.04 | -0.07, -0.01 | 0.004 |
| Key Stage 4 |  |  |  |  |  |  |  |  |
| English | 3867 | -0.12 | -0.17, -0.07 | <0.001 |  | -0.03 | -0.08, 0.01 | 0.144 |
| Math | 3867 | -0.15 | -0.19, -0.11 | <0.001 |  | -0.04 | -0.08, 0.00 | 0.043 |
| Attainment 8 | 3867 | -0.13 | -0.18, -0.09 | <0.001 |  | -0.04 | -0.08, 0.00 | 0.043 |

Linear regressions with complex survey weights were conducted. Adjusted models controlled for sex, month of birth, ethnic groups, maternal partnership status, highest household education, and household income.

**Supplementary Table S15:** Association between each 1 SD decrease in housing quality and percentage of total missed sessions in years 1 to 11 after multiple imputation in the Millennium Cohort Study.

|  | **Unadjusted** | | |  | **Adjusted** | | |
| --- | --- | --- | --- | --- | --- | --- | --- |
|  | b | 95% CI | *p* |  | b | 95% CI | *p* |
| Total absences |  |  |  |  |  |  |  |
| Year 1 | 0.73 | 0.58, 0.87 | <0.001 |  | 0.23 | 0.07, 0.40 | 0.006 |
| Year 2 | 0.67 | 0.54, 0.79 | <0.001 |  | 0.23 | 0.09, 0.37 | 0.001 |
| Year 3 | 0.63 | 0.50, 0.77 | <0.001 |  | 0.31 | 0.16, 0.46 | <0.001 |
| Year 4 | 0.48 | 0.35, 0.61 | <0.001 |  | 0.19 | 0.05, 0.34 | 0.009 |
| Year 5 | 0.43 | 0.31, 0.56 | <0.001 |  | 0.17 | 0.03, 0.32 | 0.020 |
| Year 6 | 0.35 | 0.24, 0.45 | <0.001 |  | 0.16 | 0.04, 0.28 | 0.007 |
| Year 7 | 0.48 | 0.31, 0.64 | <0.001 |  | 0.21 | 0.03, 0.39 | 0.023 |
| Year 8 | 0.45 | 0.27, 0.64 | <0.001 |  | 0.16 | -0.04, 0.36 | 0.125 |
| Year 9 | 0.22 | 0.10, 0.35 | <0.001 |  | 0.32 | 0.09, 0.56 | 0.008 |
| Year 10 | 0.81 | 0.51, 1.11 | <0.001 |  | 0.30 | -0.02, 0.62 | 0.064 |
| Year 11 | 1.04 | 0.61, 1.47 | <0.001 |  | 0.40 | 0.00, 0.79 | 0.050 |

Missing data was imputed using multiple imputations by changed regressions (10 datasets) based on all variables in the models plus auxiliary variables of housing tenure, area deprivation and employment. Linear regressions with complex survey weights were conducted. Adjusted models controlled for sex, month of birth, ethnic groups, maternal partnership status, highest household education, and household income. The sample size was n=7408.

**Supplementary Table S16**: Association between each 1 SD decrease in housing quality and school performance after multiple imputation in the Millennium Cohort Study.

|  | **Unadjusted** | | |  | **Adjusted** | | |
| --- | --- | --- | --- | --- | --- | --- | --- |
|  | β | 95% CI | *p* |  | β | 95% CI | *p* |
| Key Stage 1 |  |  |  |  |  |  |  |
| English (Reading) | -0.14 | -0.16, -0.11 | <0.001 |  | -0.03 | -0.05, 0.00 | 0.022 |
| Maths | -0.12 | -0.15, -0.10 | <0.001 |  | -0.03 | -0.05, 0.00 | 0.032 |
| Key Stage 2 |  |  |  |  |  |  |  |
| English | -0.12 | -0.15, -0.10 | <0.001 |  | -0.03 | -0.05, 0.00 | 0.060 |
| Maths | -0.12 | -0.15, -0.09 | <0.001 |  | -0.04 | -0.07, -0.01 | 0.004 |
| Key Stage 4 |  |  |  |  |  |  |  |
| English | -0.13 | -0.16, -0.10 | <0.001 |  | -0.04 | -0.06, -0.01 | 0.012 |
| Maths | -0.14 | -0.17, -0.11 | <0.001 |  | -0.04 | -0.06, -0.01 | 0.010 |
| Attainment 8 | -0.14 | -0.17, -0.11 | <0.001 |  | -0.04 | -0.07, -0.02 | 0.001 |

Missing data was imputed using multiple imputations by changed regressions (10 datasets) based on all variables in the models plus auxiliary variables of housing tenure, area deprivation and employment. Linear regressions with complex survey weights were conducted. Adjusted models controlled for sex, month of birth, ethnic groups, maternal partnership status, highest household education, and household income. The sample size was n=6861.

**Supplementary Table S17:** Associations between standardised housing conditions and percentage of total missed sessions in years 1 to 11 in the Millennium Cohort Study.

|  | **Adjusted** | | | | | | |
| --- | --- | --- | --- | --- | --- | --- | --- |
|  | b | 95% CI | *p* |  | b | 95% CI | *p* |
|  | Accommodation type | | |  | Floor level | | |
| Year 1 | 0.16 | 0.01, 0.30 | 0.041 |  | 0.09 | -0.09, 0.28 | 0.308 |
| Year 2 | 0.16 | 0.02, 0.31 | 0.029 |  | 0.13 | -0.03, 0.30 | 0.115 |
| Year 3 | 0.18 | 0.03, 0.34 | 0.020 |  | 0.11 | -0.05, 0.28 | 0.180 |
| Year 4 | 0.19 | 0.07, 0.32 | 0.003 |  | 0.07 | -0.08, 0.22 | 0.358 |
| Year 5 | 0.24 | 0.09, 0.38 | 0.002 |  | 0.14 | -0.03, 0.31 | 0.117 |
| Year 6 | 0.18 | 0.04, 0.32 | 0.012 |  | 0.06 | -0.06, 0.19 | 0.325 |
| Year 7 | 0.10 | -0.08, 0.27 | 0.273 |  | 0.09 | -0.11, 0.29 | 0.370 |
| Year 8 | 0.08 | -0.11, 0.28 | 0.405 |  | 0.07 | -0.17, 0.30 | 0.564 |
| Year 9 | 0.13 | -0.13, 0.38 | 0.328 |  | 0.12 | -0.15, 0.38 | 0.394 |
| Year 10 | 0.13 | -0.17, 0.42 | 0.400 |  | 0.18 | -0.19, 0.55 | 0.336 |
| Year 11 | 0.13 | -0.26, 0.52 | 0.509 |  | 0.25 | -0.23, 0.72 | 0.306 |
| Total | **0.15** | **0.01, 0.29** | **0.037** |  | 0.12 | -0.05, 0.29 | 0.170 |
|  | Garden access | | |  | Damp and mould | | |
| Year 1 | 0.04 | -0.11, 0.19 | 0.601 |  | 0.24 | 0.08, 0.40 | 0.004 |
| Year 2 | 0.09 | -0.05, 0.23 | 0.203 |  | 0.14 | 0.02, 0.26 | 0.020 |
| Year 3 | 0.25 | 0.07, 0.42 | 0.006 |  | 0.23 | 0.11, 0.35 | <0.001 |
| Year 4 | 0.11 | -0.04, 0.26 | 0.161 |  | 0.15 | 0.02, 0.29 | 0.025 |
| Year 5 | 0.08 | -0.07, 0.24 | 0.277 |  | 0.06 | -0.06, 0.17 | 0.329 |
| Year 6 | 0.07 | -0.07, 0.2 | 0.315 |  | 0.09 | -0.03, 0.21 | 0.127 |
| Year 7 | 0.10 | -0.1, 0.29 | 0.321 |  | 0.18 | 0.04, 0.32 | 0.013 |
| Year 8 | 0.09 | -0.12, 0.3 | 0.387 |  | 0.10 | -0.06, 0.26 | 0.209 |
| Year 9 | 0.15 | -0.12, 0.43 | 0.277 |  | 0.34 | 0.14, 0.53 | 0.001 |
| Year 10 | 0.19 | -0.11, 0.48 | 0.215 |  | 0.43 | 0.18, 0.67 | 0.001 |
| Year 11 | 0.31 | -0.09, 0.71 | 0.129 |  | 0.39 | 0.09, 0.69 | 0.012 |
| Total | 0.13 | -0.01, 0.28 | 0.076 |  | **0.21** | **0.11, 0.32** | **<0.001** |
|  | Heating | | |  | Overcrowding | | |
| Year 1 | 0.14 | 0.03, 0.26 | 0.015 |  | 0.11 | -0.03, 0.26 | 0.109 |
| Year 2 | 0.12 | -0.01, 0.26 | 0.070 |  | 0.13 | 0.00, 0.26 | 0.050 |
| Year 3 | 0.03 | -0.09, 0.15 | 0.618 |  | 0.19 | 0.06, 0.33 | 0.005 |
| Year 4 | 0.02 | -0.12, 0.15 | 0.820 |  | 0.12 | -0.02, 0.26 | 0.084 |
| Year 5 | 0.03 | -0.09, 0.14 | 0.629 |  | 0.10 | -0.02, 0.22 | 0.115 |
| Year 6 | 0.02 | -0.10, 0.13 | 0.774 |  | 0.15 | 0.04, 0.26 | 0.010 |
| Year 7 | -0.01 | -0.13, 0.11 | 0.889 |  | 0.22 | 0.06, 0.38 | 0.007 |
| Year 8 | 0.00 | -0.15, 0.15 | 0.993 |  | 0.17 | 0.03, 0.31 | 0.021 |
| Year 9 | -0.01 | -0.20, 0.18 | 0.924 |  | 0.32 | 0.12, 0.52 | 0.002 |
| Year 10 | -0.17 | -0.35, 0.02 | 0.078 |  | 0.22 | -0.08, 0.52 | 0.142 |
| Year 11 | -0.17 | -0.44, 0.11 | 0.226 |  | 0.35 | 0.04, 0.67 | 0.027 |
| Total | 0.00 | -0.10, 0.10 | 0.990 |  | **0.19** | **0.07, 0.31** | **0.002** |

Linear regressions with complex survey weights were conducted separately. Adjusted models controlled for sex, month of birth, ethnic groups, maternal partnership status, highest household education, and household income. The sample size was n=7272.

**Supplementary Table S18:** Associations between standardised housing conditions and educational attainment in the Millennium Cohort Study.

|  | **Adjusted** | | | | | | |
| --- | --- | --- | --- | --- | --- | --- | --- |
|  | b | 95% CI | *p* |  | b | 95% CI | *p* |
|  | Accommodation type | | |  | Floor level | | |
| KS1: English (Reading) | -0.01 | -0.03, 0.02 | 0.553 |  | 0.00 | -0.02, 0.03 | 0.687 |
| KS1: Math | -0.01 | -0.03, 0.02 | 0.656 |  | 0.01 | -0.02, 0.03 | 0.603 |
| KS2: English | 0.01 | -0.01, 0.04 | 0.396 |  | 0.01 | -0.01, 0.04 | 0.208 |
| KS2: Math | -0.02 | -0.05, 0.01 | 0.123 |  | -0.01 | -0.04, 0.01 | 0.345 |
| KS4: English | 0.00 | -0.03, 0.02 | 0.784 |  | 0.00 | -0.03, 0.03 | 0.823 |
| KS4: Math | -0.02 | -0.04, 0.01 | 0.253 |  | -0.01 | -0.04, 0.02 | 0.480 |
| KS4: Attainment 8 | -0.02 | -0.04, 0.01 | 0.223 |  | -0.01 | -0.04, 0.02 | 0.436 |
|  | Garden access | | |  | Damp and mould | | |
| KS1: English (Reading) | -0.01 | -0.04, 0.02 | 0.439 |  | 0.01 | -0.02, 0.03 | 0.587 |
| KS1: Math | -0.01 | -0.04, 0.01 | 0.355 |  | 0.01 | -0.01, 0.03 | 0.559 |
| KS2: English | 0.00 | -0.03, 0.02 | 0.737 |  | 0.01 | -0.01, 0.03 | 0.347 |
| KS2: Math | -0.02 | -0.05, 0.01 | 0.114 |  | 0.00 | -0.03, 0.02 | 0.725 |
| KS4: English | -0.02 | -0.05, 0.01 | 0.115 |  | 0.00 | -0.03, 0.02 | 0.714 |
| KS4: Math | -0.01 | -0.04, 0.02 | 0.366 |  | -0.01 | -0.03, 0.01 | 0.346 |
| KS4: Attainment 8 | -0.02 | -0.04, 0.01 | 0.17 |  | -0.01 | -0.03, 0.01 | 0.326 |
|  | Heating | | |  | Overcrowding | | |
| KS1: English (Reading) | -0.02 | -0.04, 0.00 | 0.098 |  | -0.06 | -0.09, -0.04 | <0.001 |
| KS1: Math | -0.02 | -0.04, 0.00 | 0.045 |  | -0.05 | -0.07, -0.03 | <0.001 |
| KS2: English | -0.02 | -0.05, 0.00 | 0.067 |  | -0.08 | -0.10, -0.05 | <0.001 |
| KS2: Math | -0.03 | -0.05, 0.00 | 0.048 |  | -0.05 | -0.08, -0.03 | <0.001 |
| KS4: English | -0.02 | -0.04, 0.00 | 0.104 |  | -0.06 | -0.09, -0.03 | <0.001 |
| KS4: Math | -0.01 | -0.04, 0.01 | 0.154 |  | -0.05 | -0.08, -0.02 | <0.001 |
| KS4: Attainment 8 | -0.02 | -0.04, 0.00 | 0.066 |  | -0.06 | -0.09, -0.04 | <0.001 |

Linear regressions with complex survey weights were conducted separately. Adjusted models controlled for sex, month of birth, ethnic groups, maternal partnership status, highest household education, and household income. The sample size was n=6741.

**Supplementary Table S19:** Associations between housing quality scale and log-transformed percentage of total missed sessions in years 1 to 11 in the Millennium Cohort Study.

|  | **Unadjusted** | | |  | **Adjusted** | | |
| --- | --- | --- | --- | --- | --- | --- | --- |
|  | b | 95% CI | *p* |  | b | 95% CI | *p* |
| Total absences |  |  |  |  |  |  |  |
| Year 1 | 0.10 | 0.09, 0.12 | <0.001 |  | 0.03 | 0.01, 0.05 | 0.001 |
| Year 2 | 0.11 | 0.09, 0.13 | <0.001 |  | 0.05 | 0.03, 0.06 | <0.001 |
| Year 3 | 0.10 | 0.08, 0.12 | <0.001 |  | 0.05 | 0.03, 0.07 | <0.001 |
| Year 4 | 0.07 | 0.05, 0.09 | <0.001 |  | 0.03 | 0.01, 0.04 | 0.011 |
| Year 5 | 0.07 | 0.05, 0.09 | <0.001 |  | 0.03 | 0.01, 0.05 | 0.007 |
| Year 6 | 0.06 | 0.04, 0.09 | <0.001 |  | 0.03 | 0.01, 0.05 | 0.001 |
| Year 7 | 0.07 | 0.05, 0.10 | <0.001 |  | 0.03 | 0.01, 0.06 | 0.005 |
| Year 8 | 0.07 | 0.04, 0.09 | <0.001 |  | 0.02 | 0.00, 0.05 | 0.037 |
| Year 9 | 0.07 | 0.05, 0.10 | <0.001 |  | 0.04 | 0.01, 0.06 | 0.010 |
| Year 10 | 0.10 | 0.07, 0.12 | <0.001 |  | 0.03 | 0.01, 0.06 | 0.018 |
| Year 11 | 0.10 | 0.09, 0.12 | <0.001 |  | 0.03 | 0.01, 0.05 | 0.001 |
| Total | 0.09 | 0.07, 0.10 | <0.001 |  | 0.03 | 0.02, 0.05 | <0.001 |

Linear regressions with complex survey weights were conducted separately. Adjusted models controlled for sex, month of birth, ethnic groups, maternal partnership status, highest household education, and household income. The sample size was n=7272.

**Supplementary Table S20:** Associations between housing quality items and log-transformed percentage of total missed sessions in years 1 to 11 in the Millennium Cohort Study.

|  | **Adjusted** | | | | | | |
| --- | --- | --- | --- | --- | --- | --- | --- |
|  | b | 95% CI | *p* |  | b | 95% CI | *p* |
|  | Accommodation type | | |  | Floor level | | |
| Year 1 | 0.09 | 0.03, 0.16 | 0.007 |  | 0.03 | -0.02, 0.08 | 0.202 |
| Year 2 | 0.15 | 0.08, 0.21 | <0.001 |  | 0.08 | 0.02, 0.13 | 0.005 |
| Year 3 | 0.10 | 0.03, 0.17 | 0.006 |  | 0.03 | -0.02, 0.08 | 0.213 |
| Year 4 | 0.11 | 0.05, 0.18 | 0.001 |  | 0.02 | -0.03, 0.07 | 0.458 |
| Year 5 | 0.15 | 0.07, 0.23 | <0.001 |  | 0.07 | 0.01, 0.14 | 0.029 |
| Year 6 | 0.13 | 0.07, 0.20 | <0.001 |  | 0.04 | -0.01, 0.09 | 0.096 |
| Year 7 | 0.09 | 0.01, 0.17 | 0.037 |  | 0.03 | -0.04, 0.10 | 0.361 |
| Year 8 | 0.06 | -0.02, 0.14 | 0.146 |  | 0.00 | -0.07, 0.07 | 0.918 |
| Year 9 | 0.10 | 0.00, 0.20 | 0.043 |  | 0.04 | -0.03, 0.12 | 0.225 |
| Year 10 | 0.10 | 0.02, 0.19 | 0.020 |  | 0.05 | -0.03, 0.13 | 0.186 |
| Year 11 | 0.09 | 0.03, 0.16 | 0.007 |  | 0.03 | -0.02, 0.08 | 0.202 |
| Total | 0.11 | 0.05, 0.16 | <0.001 |  | 0.04 | 0.00, 0.08 | 0.062 |
|  | Garden | | |  | Damp | | |
| Year 1 | 0.02 | -0.02, 0.05 | 0.341 |  | 0.05 | 0.01, 0.10 | 0.014 |
| Year 2 | 0.04 | 0.00, 0.07 | 0.026 |  | 0.04 | 0.01, 0.08 | 0.024 |
| Year 3 | 0.05 | 0.01, 0.09 | 0.007 |  | 0.08 | 0.04, 0.11 | <0.001 |
| Year 4 | 0.02 | -0.02, 0.06 | 0.344 |  | 0.05 | 0.00, 0.10 | 0.041 |
| Year 5 | 0.02 | -0.02, 0.06 | 0.377 |  | 0.01 | -0.03, 0.05 | 0.577 |
| Year 6 | 0.03 | 0.00, 0.07 | 0.077 |  | 0.02 | -0.02, 0.07 | 0.304 |
| Year 7 | 0.03 | -0.01, 0.07 | 0.146 |  | 0.05 | 0.01, 0.09 | 0.011 |
| Year 8 | 0.02 | -0.02, 0.06 | 0.414 |  | 0.04 | -0.01, 0.08 | 0.102 |
| Year 9 | 0.01 | -0.04, 0.06 | 0.577 |  | 0.08 | 0.02, 0.13 | 0.004 |
| Year 10 | 0.04 | -0.01, 0.09 | 0.098 |  | 0.06 | 0.01, 0.10 | 0.023 |
| Year 11 | 0.02 | -0.02, 0.05 | 0.341 |  | 0.05 | 0.01, 0.10 | 0.014 |
| Total | 0.03 | 0.00, 0.05 | 0.043 |  | 0.05 | 0.02, 0.08 | 0.001 |
|  | Heating | | |  | Overcrowding | | |
| Year 1 | 0.05 | 0.01, 0.1 | 0.015 |  | 0.03 | 0.00, 0.07 | 0.079 |
| Year 2 | 0.05 | 0.00, 0.09 | 0.052 |  | 0.05 | 0.01, 0.09 | 0.008 |
| Year 3 | 0.03 | -0.01, 0.07 | 0.197 |  | 0.06 | 0.02, 0.10 | 0.003 |
| Year 4 | 0.01 | -0.03, 0.06 | 0.555 |  | 0.02 | -0.01, 0.06 | 0.209 |
| Year 5 | 0.03 | -0.02, 0.08 | 0.304 |  | 0.03 | -0.01, 0.07 | 0.100 |
| Year 6 | 0.03 | -0.02, 0.08 | 0.284 |  | 0.04 | 0.01, 0.08 | 0.021 |
| Year 7 | 0.01 | -0.03, 0.06 | 0.570 |  | 0.05 | 0.01, 0.09 | 0.010 |
| Year 8 | 0.03 | -0.02, 0.08 | 0.239 |  | 0.05 | 0.01, 0.08 | 0.013 |
| Year 9 | 0.00 | -0.06, 0.05 | 0.943 |  | 0.06 | 0.02, 0.10 | 0.003 |
| Year 10 | -0.03 | -0.09, 0.03 | 0.307 |  | 0.05 | 0.00, 0.09 | 0.038 |
| Year 11 | 0.05 | 0.01, 0.10 | 0.015 |  | 0.03 | 0.00, 0.07 | 0.079 |
| Total | 0.02 | -0.01, 0.06 | 0.162 |  | 0.04 | 0.02, 0.07 | 0.001 |

Linear regressions with complex survey weights were conducted separately. Adjusted models controlled for sex, month of birth, ethnic groups, maternal partnership status, highest household education, and household income. The sample size was n=7272.

**Supplementary Table S21:** Associations between housing condition values and percentage of total missed sessions in years 1 to 11 in the Millennium Cohort Study.

|  | **Adjusted** | | | | | | |
| --- | --- | --- | --- | --- | --- | --- | --- |
|  | b | 95% CI | *p* |  | b | 95% CI | *p* |
| Accommodation type | Value 1  *(reference value ‘0’)* | | |  | Value 2  *(reference value ‘0’)* | | |
| Year 1 | 0.52 | -0.04, 1.08 | 0.070 |  | 1.56 | -1.31, 4.44 | 0.285 |
| Year 2 | 0.62 | 0.09, 1.16 | 0.021 |  | -0.15 | -2.25, 1.96 | 0.892 |
| Year 3 | 0.73 | 0.16, 1.29 | 0.012 |  | -0.61 | -2.90, 1.68 | 0.601 |
| Year 4 | 0.74 | 0.28, 1.21 | 0.002 |  | -0.28 | -1.90, 1.35 | 0.738 |
| Year 5 | 0.89 | 0.35, 1.43 | 0.001 |  | 0.12 | -2.27, 2.51 | 0.922 |
| Year 6 | 0.64 | 0.11, 1.16 | 0.017 |  | 0.99 | -0.59, 2.57 | 0.219 |
| Year 7 | 0.37 | -0.26, 1.00 | 0.242 |  | -0.27 | -2.39, 1.85 | 0.800 |
| Year 8 | 0.33 | -0.42, 1.07 | 0.391 |  | -0.16 | -2.13, 1.81 | 0.875 |
| Year 9 | 0.49 | -0.45, 1.43 | 0.302 |  | -0.36 | -3.53, 2.81 | 0.823 |
| Year 10 | 0.52 | -0.59, 1.63 | 0.358 |  | -0.82 | -2.59, 0.95 | 0.364 |
| Year 11 | 0.62 | -0.82, 2.07 | 0.397 |  | -2.80 | -5.77, 0.17 | 0.064 |
| Total | **0.59** | **0.06, 1.11** | **0.028** |  | -0.25 | -1.93, 1.43 | 0.769 |
| Floor level | Value 1  *(reference value ‘0’)* | | |  | Value 2  *(reference value ‘0’)* | | |
| Year 1 | 0.52 | -0.26, 1.30 | 0.191 |  | 0.33 | -0.62, 1.28 | 0.492 |
| Year 2 | 0.68 | -0.18, 1.53 | 0.120 |  | 0.50 | -0.36, 1.37 | 0.251 |
| Year 3 | 1.12 | 0.16, 2.08 | 0.023 |  | 0.16 | -0.58, 0.90 | 0.668 |
| Year 4 | 0.55 | -0.17, 1.27 | 0.135 |  | 0.17 | -0.67, 1.00 | 0.694 |
| Year 5 | 1.40 | 0.52, 2.27 | 0.002 |  | 0.18 | -0.65, 1.00 | 0.671 |
| Year 6 | 1.02 | 0.28, 1.76 | 0.007 |  | -0.09 | -0.72, 0.55 | 0.786 |
| Year 7 | 0.26 | -0.67, 1.19 | 0.582 |  | 0.44 | -0.55, 1.43 | 0.380 |
| Year 8 | 0.34 | -0.83, 1.51 | 0.563 |  | 0.26 | -0.92, 1.44 | 0.664 |
| Year 9 | 0.27 | -1.24, 1.78 | 0.725 |  | 0.58 | -0.62, 1.78 | 0.343 |
| Year 10 | 0.11 | -1.42, 1.64 | 0.888 |  | 1.06 | -0.99, 3.11 | 0.308 |
| Year 11 | 0.46 | -1.29, 2.21 | 0.605 |  | 1.29 | -1.40, 3.97 | 0.346 |
| Total | 0.61 | -0.19, 1.42 | 0.135 |  | 0.44 | -0.38, 1.27 | 0.289 |
| Garden access | Value 1  *(reference value ‘0’)* | | |  | Value 2  *(reference value ‘0’)* | | |
| Year 1 | 1.62 | 0.60, 2.64 | 0.002 |  | -0.03 | -0.57, 0.51 | 0.905 |
| Year 2 | 1.86 | 0.65, 3.06 | 0.003 |  | 0.13 | -0.35, 0.62 | 0.586 |
| Year 3 | 1.26 | 0.36, 2.16 | 0.006 |  | 0.79 | 0.15, 1.43 | 0.016 |
| Year 4 | 0.86 | 0.08, 1.63 | 0.031 |  | 0.31 | -0.24, 0.86 | 0.265 |
| Year 5 | 1.29 | 0.24, 2.33 | 0.016 |  | 0.17 | -0.35, 0.70 | 0.518 |
| Year 6 | 1.13 | 0.11, 2.15 | 0.030 |  | 0.13 | -0.35, 0.61 | 0.597 |
| Year 7 | 0.62 | -0.28, 1.53 | 0.174 |  | 0.30 | -0.40, 0.99 | 0.406 |
| Year 8 | 0.34 | -0.60, 1.29 | 0.472 |  | 0.31 | -0.44, 1.05 | 0.415 |
| Year 9 | 0.57 | -1.03, 2.18 | 0.482 |  | 0.51 | -0.49, 1.51 | 0.316 |
| Year 10 | -0.04 | -1.38, 1.29 | 0.951 |  | 0.71 | -0.39, 1.81 | 0.206 |
| Year 11 | 0.07 | -2.08, 2.22 | 0.949 |  | 1.15 | -0.34, 2.64 | 0.129 |
| Total | **0.87** | **0.05, 1.69** | **0.038** |  | 0.41 | -0.13, 0.94 | 0.135 |
| Damp | Value 1  *(reference value ‘0’)* | | |  | Value 2  *(reference value ‘0’)* | | |
| Year 1 | 0.14 | -0.22, 0.5 | 0.435 |  | 1.88 | 0.67, 3.08 | 0.002 |
| Year 2 | 0.04 | -0.26, 0.34 | 0.783 |  | 1.22 | 0.31, 2.13 | 0.009 |
| Year 3 | 0.20 | -0.11, 0.51 | 0.197 |  | 1.69 | 0.87, 2.52 | 0.000 |
| Year 4 | 0.27 | -0.05, 0.59 | 0.102 |  | 0.84 | -0.06, 1.74 | 0.068 |
| Year 5 | 0.10 | -0.24, 0.44 | 0.579 |  | 0.30 | -0.37, 0.98 | 0.375 |
| Year 6 | 0.26 | -0.10, 0.63 | 0.156 |  | 0.28 | -0.53, 1.09 | 0.498 |
| Year 7 | 0.30 | -0.13, 0.73 | 0.167 |  | 0.99 | 0.05, 1.92 | 0.039 |
| Year 8 | 0.13 | -0.27, 0.53 | 0.534 |  | 0.66 | -0.50, 1.81 | 0.264 |
| Year 9 | 0.70 | 0.01, 1.39 | 0.048 |  | 1.60 | 0.47, 2.72 | 0.006 |
| Year 10 | 1.18 | 0.36, 2.00 | 0.005 |  | 1.41 | -0.20, 3.02 | 0.086 |
| Year 11 | 0.86 | -0.14, 1.87 | 0.092 |  | 1.74 | -0.42, 3.91 | 0.113 |
| Total | **0.38** | **0.03, 0.73** | **0.031** |  | **1.15** | **0.38, 1.91** | **0.003** |
| Heating |  | | |  | Value 2  *(reference value ‘0’)* | | |
| Year 1 |  |  |  |  | 0.73 | 0.14, 1.32 | 0.015 |
| Year 2 |  |  |  |  | 0.62 | -0.05, 1.30 | 0.070 |
| Year 3 |  |  |  |  | 0.15 | -0.44, 0.74 | 0.618 |
| Year 4 |  |  |  |  | 0.08 | -0.60, 0.76 | 0.820 |
| Year 5 |  |  |  |  | 0.14 | -0.44, 0.72 | 0.629 |
| Year 6 |  |  |  |  | 0.09 | -0.51, 0.68 | 0.774 |
| Year 7 |  |  |  |  | -0.04 | -0.65, 0.56 | 0.889 |
| Year 8 |  |  |  |  | 0.00 | -0.76, 0.76 | 0.993 |
| Year 9 |  |  |  |  | -0.05 | -1.00, 0.91 | 0.924 |
| Year 10 |  |  |  |  | -0.84 | -1.77, 0.09 | 0.078 |
| Year 11 |  |  |  |  | -0.85 | -2.23, 0.53 | 0.226 |
| Total |  |  |  |  | 0.00 | -0.52, 0.52 | 0.990 |
| Overcrowding | Value 1  *(reference value ‘0’)* | | |  | Value 2  *(reference value ‘0’)* | | |
| Year 1 | 0.00 | -0.29, 0.29 | 0.998 |  | 0.49 | -0.08, 1.06 | 0.092 |
| Year 2 | 0.13 | -0.14, 0.40 | 0.357 |  | 0.52 | 0.00, 1.04 | 0.049 |
| Year 3 | 0.03 | -0.26, 0.32 | 0.849 |  | 0.82 | 0.28, 1.35 | 0.003 |
| Year 4 | -0.10 | -0.38, 0.17 | 0.457 |  | 0.55 | 0.00, 1.09 | 0.049 |
| Year 5 | 0.10 | -0.17, 0.38 | 0.462 |  | 0.38 | -0.09, 0.84 | 0.111 |
| Year 6 | 0.16 | -0.10, 0.42 | 0.241 |  | 0.58 | 0.14, 1.02 | 0.010 |
| Year 7 | 0.02 | -0.31, 0.35 | 0.918 |  | 0.94 | 0.30, 1.58 | 0.004 |
| Year 8 | 0.09 | -0.25, 0.43 | 0.596 |  | 0.69 | 0.13, 1.25 | 0.016 |
| Year 9 | 0.17 | -0.29, 0.63 | 0.461 |  | 1.30 | 0.52, 2.08 | 0.001 |
| Year 10 | -0.20 | -0.90, 0.49 | 0.565 |  | 1.01 | -0.13, 2.14 | 0.081 |
| Year 11 | -0.05 | -0.73, 0.63 | 0.881 |  | 1.52 | 0.25, 2.78 | 0.019 |
| Total | 0.03 | -0.24, 0.30 | 0.824 |  | **0.80** | **0.32, 1.27** | **0.001** |

Linear regressions with complex survey weights were conducted separately. Adjusted models controlled for sex, month of birth, ethnic groups, maternal partnership status, highest household education, and household income. Reference values were the positive housing conditions (i.e. value ‘0’), associations for intermediate (value ‘1’) and negative (value ‘2’) features from the same models are shown. The sample size was n=7272.

**Supplementary Table S22:** Associations between housing condition values and educational attainment in the Millennium Cohort Study.

|  | **Adjusted** | | | | | | |
| --- | --- | --- | --- | --- | --- | --- | --- |
|  | b | 95% CI | *p* |  | b | 95% CI | *p* |
| Accommodation type | Value 1  *(reference value ‘0’)* | | |  | Value 2  *(reference value ‘0’)* | | |
| KS1: English (Reading) | 0.00 | -0.09, 0.08 | 0.971 |  | -0.57 | -1.30, 0.16 | 0.124 |
| KS1: Math | 0.00 | -0.09, 0.09 | 0.938 |  | -0.40 | -1.11, 0.31 | 0.269 |
| KS2: English | 0.05 | -0.03, 0.14 | 0.198 |  | -0.26 | -1.00, 0.47 | 0.481 |
| KS2: Math | -0.06 | -0.16, 0.03 | 0.191 |  | -0.44 | -1.28, 0.40 | 0.301 |
| KS4: English | 0.00 | -0.11, 0.10 | 0.925 |  | -0.20 | -0.71, 0.31 | 0.439 |
| KS4: Math | -0.04 | -0.14, 0.06 | 0.407 |  | -0.42 | -1.11, 0.28 | 0.236 |
| KS4: Attainment 8 | -0.04 | -0.13, 0.04 | 0.317 |  | -0.28 | -0.77, 0.22 | 0.275 |
| Floor level | Value 1  *(reference value ‘0’)* | | |  | Value 2  *(reference value ‘0’)* | | |
| KS1: English (Reading) | -0.01 | -0.13, 0.12 | 0.897 |  | 0.03 | -0.10, 0.16 | 0.625 |
| KS1: Math | -0.07 | -0.21, 0.07 | 0.334 |  | 0.07 | -0.04, 0.18 | 0.235 |
| KS2: English | -0.03 | -0.17, 0.11 | 0.665 |  | 0.10 | -0.02, 0.23 | 0.098 |
| KS2: Math | -0.14 | -0.27, -0.01 | 0.034 |  | -0.01 | -0.15, 0.13 | 0.869 |
| KS4: English | -0.14 | -0.28, 0.00 | 0.052 |  | 0.04 | -0.11, 0.20 | 0.582 |
| KS4: Math | -0.10 | -0.23, 0.02 | 0.102 |  | -0.01 | -0.17, 0.14 | 0.873 |
| KS4: Attainment 8 | -0.13 | -0.24, -0.01 | 0.029 |  | -0.01 | -0.16, 0.14 | 0.947 |
| Garden access | Value 1  *(reference value ‘0’)* | | |  | Value 2  *(reference value ‘0’)* | | |
| KS1: English (Reading) | 0.00 | -0.11, 0.1 | 0.925 |  | -0.20 | -0.71, 0.31 | 0.439 |
| KS1: Math | -0.15 | -0.32, 0.01 | 0.063 |  | -0.02 | -0.11, 0.06 | 0.578 |
| KS2: English | 0.02 | -0.16, 0.20 | 0.817 |  | -0.02 | -0.13, 0.08 | 0.695 |
| KS2: Math | -0.13 | -0.29, 0.04 | 0.127 |  | -0.07 | -0.17, 0.03 | 0.181 |
| KS4: English | -0.08 | -0.23, 0.08 | 0.329 |  | -0.07 | -0.17, 0.02 | 0.146 |
| KS4: Math | -0.09 | -0.22, 0.05 | 0.226 |  | -0.04 | -0.14, 0.06 | 0.454 |
| KS4: Attainment 8 | -0.10 | -0.22, 0.03 | 0.128 |  | -0.05 | -0.14, 0.04 | 0.242 |
| Damp | Value 1  *(reference value ‘0’)* | | |  | Value 2  *(reference value ‘0’)* | | |
| KS1: English (Reading) | 0.05 | -0.02, 0.12 | 0.171 |  | -0.04 | -0.20, 0.12 | 0.622 |
| KS1: Math | 0.03 | -0.03, 0.10 | 0.305 |  | -0.02 | -0.15, 0.12 | 0.815 |
| KS2: English | 0.02 | -0.05, 0.10 | 0.551 |  | 0.05 | -0.10, 0.21 | 0.483 |
| KS2: Math | 0.05 | -0.02, 0.12 | 0.167 |  | -0.14 | -0.30, 0.02 | 0.076 |
| KS4: English | 0.02 | -0.05, 0.10 | 0.581 |  | -0.09 | -0.22, 0.05 | 0.207 |
| KS4: Math | 0.01 | -0.05, 0.08 | 0.746 |  | -0.12 | -0.25, 0.00 | 0.059 |
| KS4: Attainment 8 | 0.00 | -0.06, 0.06 | 0.996 |  | -0.10 | -0.22, 0.02 | 0.112 |
| Heating |  | | |  | Value 2  *(reference value ‘0’)* | | |
| KS1: English (Reading) |  |  |  |  | -0.09 | -0.19, 0.02 | 0.098 |
| KS1: Math |  |  |  |  | -0.11 | -0.22, 0.00 | 0.045 |
| KS2: English |  |  |  |  | -0.11 | -0.23, 0.01 | 0.067 |
| KS2: Math |  |  |  |  | -0.13 | -0.27, 0.00 | 0.048 |
| KS4: English |  |  |  |  | -0.09 | -0.21, 0.02 | 0.104 |
| KS4: Math |  |  |  |  | -0.08 | -0.18, 0.03 | 0.154 |
| KS4: Attainment 8 |  |  |  |  | -0.09 | -0.19, 0.01 | 0.066 |
| Overcrowding | Value 1  *(reference value ‘0’)* | | |  | Value 2  *(reference value ‘0’)* | | |
| KS1: English (Reading) | -0.12 | -0.17, -0.06 | <0.001 |  | -0.23 | -0.32, -0.14 | <0.001 |
| KS1: Math | -0.09 | -0.14, -0.04 | 0.001 |  | -0.20 | -0.29, -0.11 | <0.001 |
| KS2: English | -0.14 | -0.20, -0.08 | <0.001 |  | -0.29 | -0.39, -0.19 | <0.001 |
| KS2: Math | -0.10 | -0.16, -0.03 | 0.003 |  | -0.19 | -0.29, -0.10 | <0.001 |
| KS4: English | -0.11 | -0.17, -0.05 | <0.001 |  | -0.22 | -0.32, -0.11 | <0.001 |
| KS4: Math | -0.10 | -0.16, -0.04 | 0.002 |  | -0.18 | -0.28, -0.09 | <0.001 |
| KS4: Attainment 8 | -0.10 | -0.16, -0.05 | <0.001 |  | -0.24 | -0.33, -0.15 | <0.001 |

Linear regressions with complex survey weights were conducted separately. Adjusted models controlled for sex, month of birth, ethnic groups, maternal partnership status, highest household education, and household income. Reference values were the positive housing conditions (i.e. value ‘0’), associations for intermediate (value ‘1’) and negative (value ‘2’) features from the same models are shown. The sample size was n=6741.

**Supplementary Table S23**: Associations between housing quality scale, housing conditions and percentage of missed sessions across compulsory education using two methods of pooling across years in the Millennium Cohort Study.

|  | **Pooled linear regression** | | |  | **Linear mixed-effects regression** | | |
| --- | --- | --- | --- | --- | --- | --- | --- |
|  | b | 95% CI | *p* |  | b | 95% CI | *p* |
| Total absences |  |  |  |  |  |  |  |
| Housing quality score | 0.24 | 0.10, 0.38 | 0.001 |  | 0.15 | 0.05, 0.24 | 0.003 |
| Accommodation type | 0.53 | 0.03, 1.02 | 0.037 |  | 0.17 | -0.17, 0.50 | 0.334 |
| Floor level | 0.30 | -0.13, 0.72 | 0.170 |  | 0.08 | -0.16, 0.31 | 0.533 |
| Garden access | 0.24 | -0.02, 0.50 | 0.076 |  | 0.12 | -0.05, 0.29 | 0.171 |
| Damp | 0.47 | 0.24, 0.71 | <0.001 |  | 0.38 | 0.18, 0.59 | <0.001 |
| Heating | 0.00 | -0.26, 0.26 | 0.990 |  | 0.01 | -0.22, 0.24 | 0.939 |
| Overcrowding | 0.35 | 0.13, 0.58 | 0.002 |  | 0.27 | 0.08, 0.46 | 0.006 |
| Authorised absences |  |  |  |  |  |  |  |
| Housing quality score | 0.12 | 0.04, 0.21 | 0.005 |  | 0.07 | 0.00, 0.14 | 0.057 |
| Unauthorised absences |  |  |  |  |  |  |  |
| Housing quality score | 0.12 | 0.05, 0.19 | 0.001 |  | 0.08 | 0.03, 0.13 | 0.001 |

Pooled linear regressions and linear mixed-effects regression with complex survey weights were conducted separately for each item and the total scores. Models were controlled for sex, month of birth, ethnic groups, maternal partnership status, highest household education, and household income. The sample size was n=7272, with a total of 79992 observations.
